# Circadian Genes MBOAT2/CDA/LPCAT2/B4GALT5 in the Metabolic Pathway Serve as New Biomarkers of PACA Prognosis and Immune Infiltration

**DOI:** 10.3390/life13051116

**Published:** 2023-04-30

**Authors:** Qingqing Wang, Shuning Zhou, Xinyi Hu, Xianggang Wang, Xue Wu, Ziyou Huai, Yu Gao, Shujing Li

**Affiliations:** 1 School of Life Science, Bengbu Medical College, Bengbu 233030, China; 2 Anhui Province Key Laboratory of Translational Cancer Research, Bengbu Medical College, Bengbu 233030, China

**Keywords:** pancreatic cancer, circadian rhythm, immune infiltration, MBOAT2, CDA, LPCAT2, B4GALT5

## Abstract

**Simple Summary:**

In this study, we found 299 circadian genes that were differentially expressed in pancreatic cancer (PACA). The expression levels of MBOAT2, CDA, LPCAT2 and B4GALT5, which were enriched in metabolic pathways, were not only highly connected with overall survival and other clinical parameters in PACA patients, but were also closely correlated with the level of immune cell infiltration. In conclusion, there was a close link between circadian genes, cancer marker pathways and immune infiltration. These findings could lay the foundation for the use of personalized chronotherapy and immune checkpoint inhibitors in the treatment of pancreatic cancer.

**Abstract:**

Pancreatic cancer (PACA) is a highly malignant tumor with a poor prognosis. Recent studies have discovered substantial differences in the expression levels of several circadian genes in PACA samples compared to normal samples. The goal of this research was to find differentially expressed rhythm genes (DERGs) in PACA samples and determine their role in the development of PACA. A total of 299 DERGs were identified in PACA, including 134 downregulated genes and 165 upregulated genes. DERGs were significantly abundant in the metabolic pathway and immune response pathways, according to GO and KEGG analyses. Survival analyses showed that PACA patients who had higher expression levels of MBOAT2/CDA/LPCAT2/B4GALT5 had shorter overall survival times. Using cell assay verification, the mRNA levels of MBOAT2/CDA/LPCAT2/B4GALT5 in Patu-8988 and PNAC-1 cells were found to be significantly higher than those in HPDE6-C7 cells, which was in line with previous studies on PACA patient data. Through conducting univariate Cox analysis, it was determined that MBOAT2/CDA/LPCAT2/B4GALT5 expression, age and grade were all high-risk factors. The MBOAT2/CDA/LPCAT2/B4GALT5 genes were independently correlated with overall survival, according to the multivariate Cox analysis. The proportion of immune cells in PACA and normal samples significantly changed, according to the immune infiltration analysis. Furthermore, MBOAT2/CDA/LPCAT2/B4GALT5 expression levels were significantly related to the level of immune cell infiltration. The protein–protein interaction network of the MBOAT2/CDA/LPCAT2/B4GALT5 genes included 54 biological nodes and 368 interacting genes. In conclusion, the finding of these DERGs adds to the investigation of the molecular processes underlying the onset and progression of PACA. In the future, DERGs may serve as prognostic and diagnostic biomarkers as well as drug targets for chronotherapy in PACA patients.

## 1. Introduction

Pancreatic cancer is a type of cancerous tumor that has a poor prognosis and high death rate. It is one of the most deadly cancerous tumors. Since the 1960s, survival rates for other major cancers have significantly increased, but survival rates for pancreatic cancer have remained relatively unchanged [1]. Patients with pancreatic cancer have insidious early symptoms, and more than 80% patients have progressed to advanced stages by the time they are seen, missing the opportunity for a surgical cure. Patients who have successfully undergone surgery for pancreatic cancer are also highly susceptible to metastasis, with a 5-year survival rate of only 15% to 20% [2,3]. Unlike other cancer types, radiotherapy and immunotherapy for pancreatic cancer are very inefficient, and research on molecular typing and precise treatment techniques is relatively underdeveloped, so there is an urgent need for the development of new drugs or treatments to improve patient prognoses [4]. In addition, the molecular mechanisms underlying the development or progression of pancreatic cancer remain unclear.

Circadian rhythms exist in most mammalian physiological processes and behaviors to enable mammals to adapt to the 24 h light–dark cycle of the Earth’s rotation, which is driven by internal timing systems and external environmental signals (light, temperature, etc.). Circadian clocks enable living organisms to anticipate changes in the external environment, align their life activities with the external environment, use resources more efficiently and avoid natural enemies. Thus, circadian clocks help living organisms to survive. To achieve this survival advantage, mammalian cells contain a core set of circadian genes that interact to form a cell-autonomous regulatory network of transcriptional oscillations capable of regulating the expression of many other genes critical to cell physiology and metabolism. Circadian regulation is very common, with nearly half of mammalian genes being rhythmically expressed in one or more tissues. In recent years, people have increasingly recognized the importance of circadian genes and circadian rhythm regulation for health. Circadian genes are not only involved in sleep disorders and mental illness, but also in the development of metabolic diseases, cardiovascular diseases and cancer [5]. Interestingly, bioinformatics analysis can help us identify differentially expressed rhythm genes, whose expression levels are closely related to the age, sex, stage, and overall survival of cancer patients as molecular markers of tumors. Previous studies have successfully identified differentially expressed rhythm genes in hepatocellular carcinoma, glioma and kidney renal clear cell carcinoma [6,7,8], but little is known about differentially expressed rhythm genes in pancreatic cancer.

In this study, our goal was to utilize information from the GTEx, TCGA and CGDB databases to identify differentially expressed rhythm genes using well-established bioinformatics methods. This study may benefit the exploration of the underlying mechanisms involved in pancreatic cancer growth and progression, and it may be utilized as a future biomarker for the prognosis, diagnosis and temporal therapy of pancreatic cancer patients.

## 2. Materials and Methods

**Figure 1:**
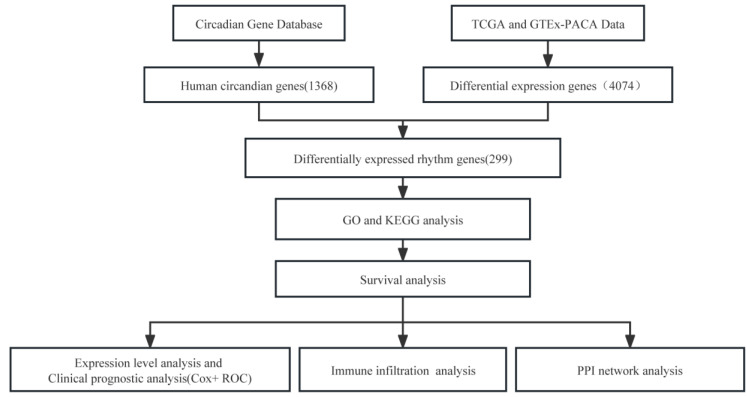
The flowchart for this study.

The flow diagram of this study is depicted in Figure 1. We downloaded original sample data from the TCGA, GTEx and CGDB databases and used bioinformatics methods to identify differentially expressed rhythm genes in pancreatic cancer and carried out GO and KEGG pathway analyses, survival analysis, clinical prognostic analysis, expression level analysis, immune infiltration analysis and PPI network analysis using the data. Moreover, experimental validation was conducted at the cellular level.

### 2.1. DERGs Identification

The Cancer Genome Atlas (TCGA; https://portal.gdc.cancer.gov/, access date: 31 May 2022) database was used to retrieve data from 174 PACA tumor samples and 4 non-tumor samples. To compensate for the lack of non-tumor samples in the TCGA database, 167 non-tumor samples were downloaded from the Gene Tissue Expression database (GTEx; http://xena.ucsc.edu/, access date: 18 May 2016). We screened the gene expression data using R software and found 4074 differentially expressed genes (DEGs, |Log2(Fold Change)| > 1 and P.adjust < 0.05). We compared 4074 DEGs with 1368 human rhythmic genes (CGDB; http://cgdb.biocuckoo.org/index.php, access date: 31 October 2020) and found 165 upregulated DERGs and 134 downregulated DERGs using the Venny 2.1 web tool (https://bioinfogp.cnb.csic.es/tools/venny/index.html, access date:16 April 2022). The heatmap package was used to create the DERG heat map.

### 2.2. GO and KEGG Pathway Analyses

For GO analysis, we computed the |Log2(Fold Change)| > 2 threshold value of the mRNA level difference using the limma package of the R software. The package ggplot2 of R software was then used to conduct functional enrichment analyses. We used an online website for KEGG pathway analysis to create bubble maps (http://www.bioinformatics.com.cn/plot_basic_hbar_with_pvalue_color_plot_105, access date:24 December 2022). P.adjust < 0.05 was considered statistically significant.

### 2.3. Survival Analysis

We retrieved 174 PACA tumor samples from the TCGA database. Individuals with incomplete clinical data were removed according to their age, TNM stage, depth of invasion, lymph node metastases and other clinicopathologic factors, yielding a total of 167 patient samples. The median gene expression of 167 PACA samples was utilized as the cut-off value. The patient samples were sorted into two groups: high and low. We performed an overall survival (OS; overall survival is defined as the time from randomization until death from any cause and is measured in the intent-to-treat population) study for 36 DERGs in metabolic pathways using the survminer and survival R packages. The survival curves of MBOAT2, CDA, LPCAT2 and B4GALT5 genes were then validated using Kaplan–Meier plotter online tools (https://kmplot.com/analysis/, access date: 5 March 2023).

### 2.4. Cell Experiment

HPDE6-C7 (EK-Bioscience Inc., STR-Identified, Shanghai, China), Patu-8988 (iCell Bioscience Inc., STR-Identified, Shanghai, China) and PNAC-1 cells (Procell Inc., STR-Identified, Wuhan, China) were cultured in DMEM (Procell, China) supplemented with 10% fetal calf serum (Gibco, USA) and 1% penicillin and streptomycin (Biosharp, China) at 37 °C in 5% CO_2_. In qRT-PCR studies, the total RNA was extracted from cells. The primers were as follows: MBOAT2-fw: gcccatcgaccaggtatgc; MBOAT2-rv: cagcgagctgctccattttc; CDA-fw: caattgctatcgccagtgaca; CDA-rv: ccatccggcttggtcatgta; LPCAT2-fw: cctctggttggcagactgtt; LPCAT2-rv: tcacagtatcctggggccat; B4GALT5-fw: ctcgctgctgtacttcgtct; B4GALT5-rv: taaggatcgccaccttccac.

### 2.5. Clinical Prognostic Analysis

To assess the combined influence of clinical characteristics and gene expression in these 167 patient samples, the R survival package was used to perform the univeriate Cox regression and multivariate Cox regression analyses [9,10]. MBOAT2, CDA, LPCAT2 and B4GALT5 prognostic models were tested using receiver operating characteristics (ROC) curves and area under the curve (AUC) analysis.

### 2.6. Immune Infiltration Assay

The cybersort package of the R program was used to determine how many of the 22 different types of functional immune cells are present in PACA and how they relate to each other. We used a website for online analysis (https://cistrome.shinyapps.io/timer/, access date:14 October 2021) to find DERGs and immune cells that had infiltrated into the tumor. In addition, we utilized TIMER 2.0 for correlation analyses to identify the association between MBOAT2/CDA/LPCAT2/B4GALT and the gene biomarkers of immune cells.

### 2.7. PPI Protein Network Interaction Analysis

STRING is a bioinformatics database that uses known and expected protein–protein interactions (PPIs) to build a PPI network of DERGs. After using the software’s results to determine how the proteins interact, we used the Cytoscape program (version 3.9.0) to visualize the PPI network.

### 2.8. Statistical Analysis

R software was used to perform data processing (version 4.0.3). The two data sets were compared using Mann–Whitney and *t* tests. *p* < 0.05 or P.adjust < 0.05 was deemed statistically significant. The standard deviation (SD) of the mean is indicated by the error bars.

## 3. Results

### 3.1. DERGs Identification

**Figure 2:**
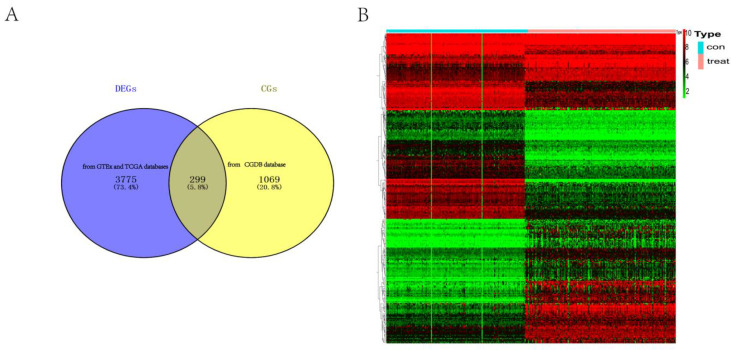
Identification of DERGs. (**A**) Intersection map of DEGs (from GTEx and TCGA databases) and CGs (from CGDB database); (**B**) heat map of DERGs. The red and green colors represent upregulated and downregulated genes, respectively.

When the GTEx and TCGA databases were used together, a total of 4074 genes with different levels of expression were identified. We discovered 299 differently expressed rhythm genes, of which 134 were downregulated and 165 were upregulated, by intersecting the 4074 differentially expressed genes (DEGs) with the 1368 circadian genes (CGs) from the CGDB database (Figure 2 and Table S1).

### 3.2. GO and KEGG Analysis

**Figure 3:**
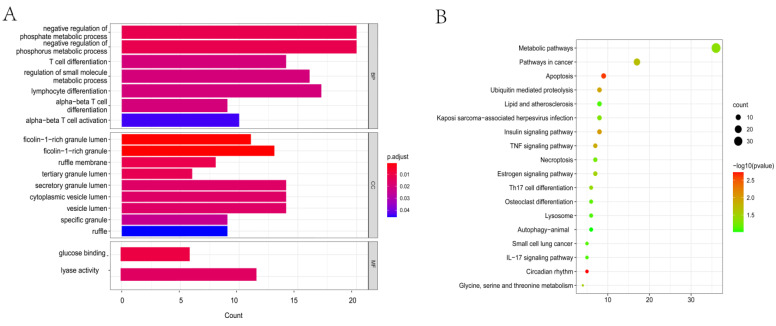
GO and KEGG analyses of DERGs (P.adjust < 0.05). (**A**) Biological process, cell components and molecular function enrichment analyses of DERGs; (**B**) KEGG pathway enrichment analysis of DERGs.

To further evaluate the function of the differentially expressed rhythm genes, we performed GO and KEGG analyses. GO enrichment analysis of BP terms showed that the DERGs were mainly enriched in the negative regulation of phosphate metabolic processes, T cell differentiation, and other processes. GO enrichment analysis of CC terms indicated that the DERGs were mainly enriched in the ficolin-1-rich granule lumen process. GO enrichment analysis of MF terms indicated that the DERGs were enriched in the glucose binding and lyase activity processes (Figure 3A). The KEGG pathway enrichment analysis showed that DERGs were significantly enriched in the circadian pathway, apoptosis, insulin signaling pathway, tumor necrosis factor signaling pathway and metabolic pathways. Among them, 36 DERGs were significantly enriched in the metabolic pathways (Figure 3B).

### 3.3. Survival Analysis

**Figure 4:**
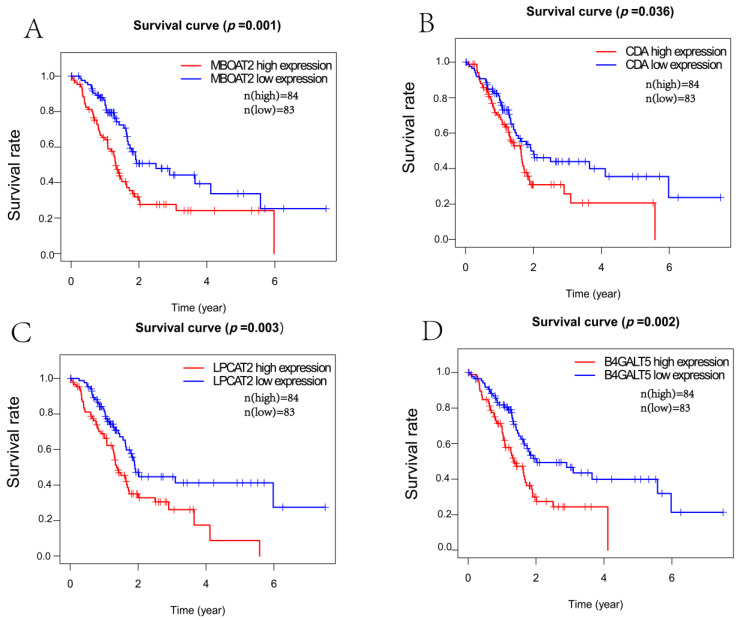
Survival prognostic analysis between the expression levels of DERGs and the survival rate of PACA patients. (**A**) MBOAT2; (**B**) CDA; (**C**) LPCAT2; (**D**) B4GALT5.

We examined the overall survival rate of 167 PACA patients to determine the predictive relevance of DERGs enriched in metabolic pathways. Higher expression levels of MBOAT2, CDA, LPCAT2 and B4GALT5 were observed to be notably linked with poorer overall survival rates in PACA patients (Figure 4). KM Plotter, an online application, was used to further confirm these results (Figure S1). In contrast, the expression levels of the remaining 32 DERGs enriched in the metabolic pathways showed no meaningful link with the overall survival rate of PACA patients. The findings revealed that the MBOAT2, CDA, LPCAT2 and B4GALT genes could be predictive factors.

### 3.4. Expression Level Analysis

**Figure 5:**
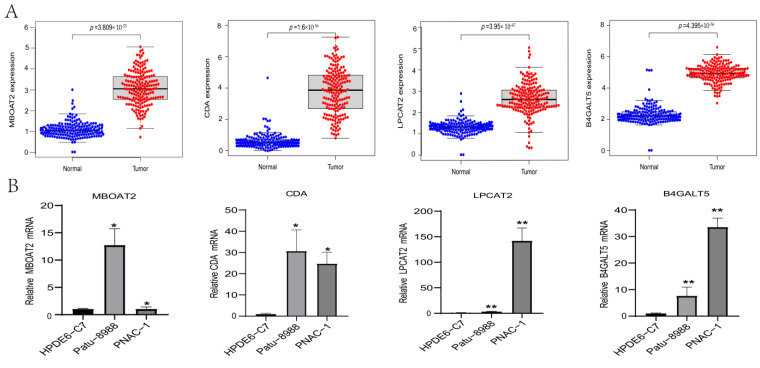
The expression levels of DERGs. (**A**) MBOAT2, CDA, LPCAT2 and B4GALT5 expression levels in normal and PACA samples; (**B**) the mRNA expression levels of MBOAT2, CDA, LPCAT2 and B4GALT5 in PACA cell lines (Patu−8988 and PNAC−1) and control cells (HPDE6−C7), as determined using qRT-PCR (*n* ≥ 3). Student’s *t* test. ** *p* < 0.01, * *p* < 0.05.

We analyzed the mRNA expression levels of the MBOAT2/CDA/LPCAT2/B4GALT5 genes in PACA patient samples and pancreatic cancer cells (Table S2). The results show that the mRNA levels of the MBOAT2/CDA/LPCAT2/B4GALT5 genes were higher in PACA samples than in normal samples (Figure 5A). GEPIA online analysis showed the same results (Figure S2). We also validated our findings in cell lines. In line with our findings, the mRNA levels of the MBOAT2/CDA/LPCAT2/B4GALT5 genes were considerably higher in Patu−8988 and PNAC−1 cells than in HPDE6−C7 cells (Figure 5B). It is proposed that MBOAT2, CDA, LPCAT2 and B4GALT5 expression may promote the onset and progression of pancreatic cancer.

### 3.5. Clinical Prognostic Analysis

**Figure 6:**
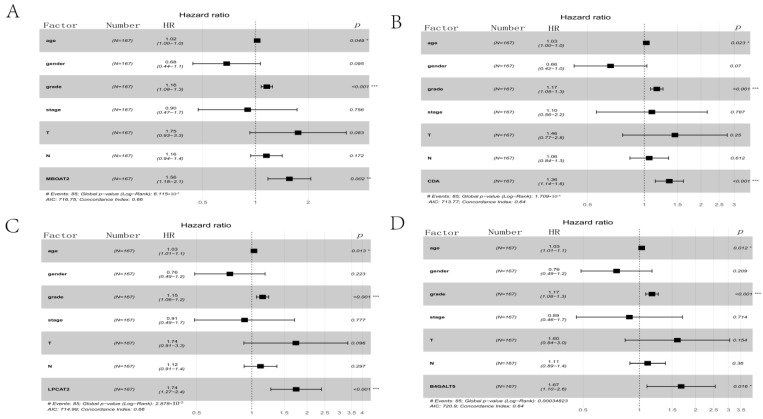
Multivariate Cox analysis of DERGs. (**A**) MBOAT2; (**B**) CDA; (**C**) LPCAT2; (**D**) B4GALT5. *** *p* < 0.001, ** *p* < 0.01 and * *p* < 0.05.

To study MBOAT2/CDA/LPCAT2/B4GALT5 expression levels and clinical relevance, clinical data from 167 PACA patients in the TCGA database were used to conduct a Cox analysis and a receiver operating characteristic (ROC) curve analysis for the DERGs (Table S3). Univariate Cox analyses revealed that age, grade, MBOAT2 expression, CDA expression, LPCAT2 expression and B4GALT5 expression were high-risk factors (Table 1). Multivariate Cox analyses indicated that the expression levels of MBOAT2, CDA, LPCAT2 and B4GALT5 were also independently associated with the overall survival rate of PACA patients (Figure 6). Meanwhile, the ROC curves were used to evaluate the sensitivity and specificity of the constructed prognostic risk features. MBOAT2, CDA, LPCAT2 and B4GALT5 had AUC values of 0.697, 0.695, 0.645 and 0.613, respectively (Figure S3). Based on these findings, the risk models show good predictive potential and the circadian genes MBOAT2/CDA/LPCAT2/B4GALT5 were adverse prognostic factors and independent prognostic indicators.

### 3.6. Immune Infiltration Analysis

**Figure 7:**
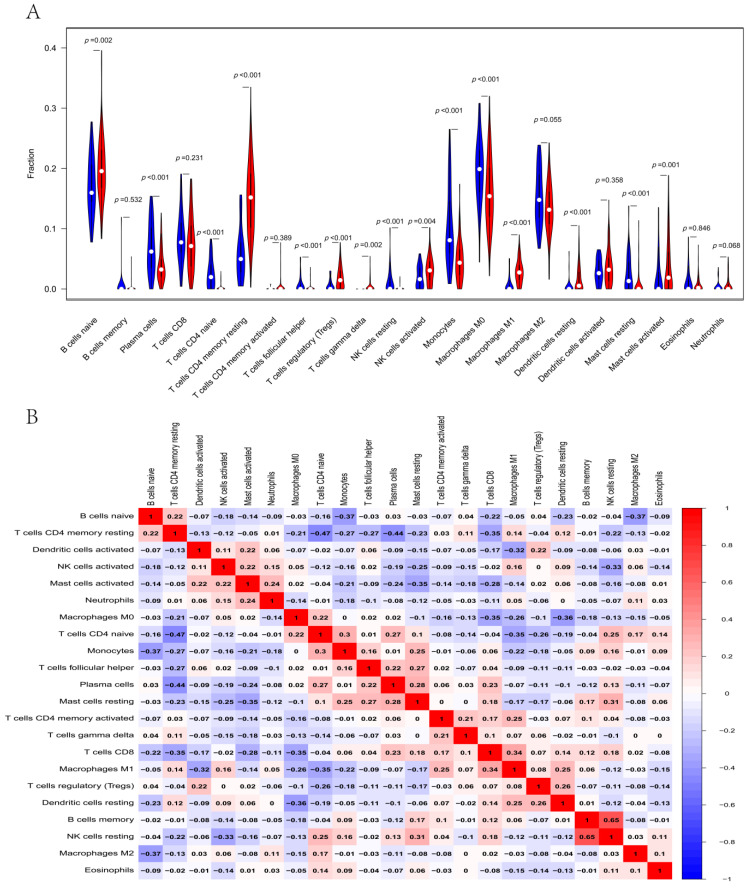
Immune infiltration analysis of DERGs. (**A**) Violin diagram visualizing infiltrated immune cells. The blue and red plot represent paired normal and tumor tissues, respectively; (**B**) correlation heat maps indicating correlations between infiltrated immune cells in PACA.

Analyses of GO terms indicated a significant relationship between DERGs and immune response function. Thus, we carried out a study of immune infiltration. The results showed that the proportion of immune cells in pancreatic cancer tissues was significantly different from that in normal tissues. Compared with paired normal tissues, pancreatic cancer tissues had more naive B cells, resting CD4 memory T cells, regulatory T cells (Tregs), activated NK cells, M1macrophages, resting dendritic cells, and activated mast cells, and fewer plasma cells, naive CD4 T cells, follicular helper T cells, resting NK cells, monocytes, M0 macrophages and resting mast cells (Figure 7A). Resting CD4 memory T cells were negatively correlated with plasma cells (Pearson correlation = −0.44), while resting mast cells were positively correlated with resting NK cells (Pearson correlation = 0.31) (Figure 7B). Moreover, the expression of MBOAT2 was negatively correlated with the infiltration of CD4+ T cells and favorably correlated with the infiltration of CD8+ T cells and dendritic cells. The expression of CDA was positively correlated with the infiltration of neutrophils and dendritic cells. LPCAT2 expression was positively correlated with the infiltration of B cells, CD8+ T cells and so on. B4GALT5 expression was positively correlated with the infiltration of B cells, CD8+ T cells, dendritic cells and so on (Figure S4). The results indicate a connection between circadian gene expression and immune cell infiltration in pancreatic cancer.

### 3.7. PPI Network Analysis

**Figure 8:**
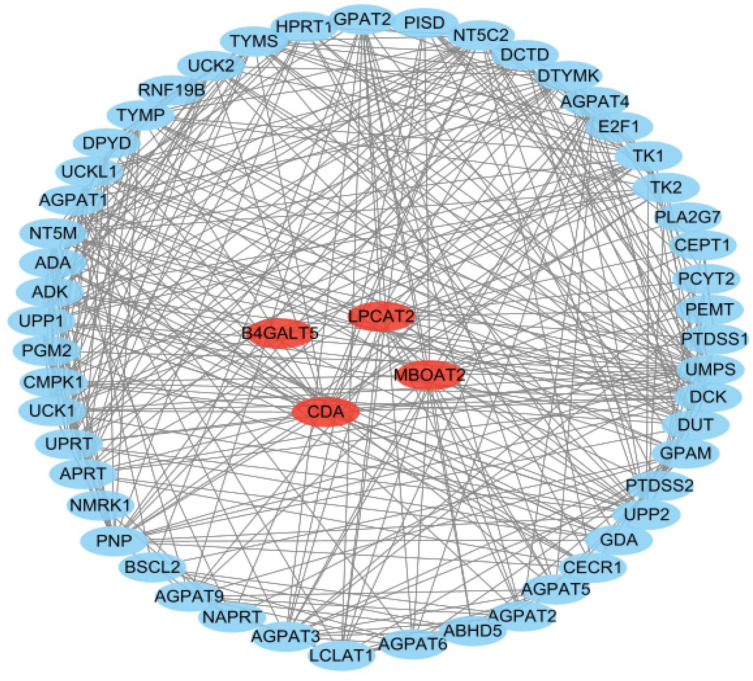
PPI network construction. Four key genes are marked in red. The interacting genes are marked in blue.

To discover significant genes and critical gene modules, we constructed a PPI network of the MBOAT2, CDA, LPCAT2 and B4GALT5 genes with 54 biological nodes and 368 interacting genes. Among them, ADA, GDA, DCK, TYMS, UCKL1, PNP, APRT, AGPAT2, LPCAT2, GPAM, MBOAT2, AGPAT6, AGPAT9, TK1, DTYMK UPP1, UCK2, DPYD, UPRT, TYMP, PEMT and PISD were the most enriched genes in the metabolic pathways (Figure 8).

## 4. Discussion

Epidemiological studies have demonstrated that disruption to circadian rhythms stimulates the proliferation of cancer cells in the tumor microenvironment and affects the levels of key hormones (such as melatonin, estrogen, etc.). Furthermore, studies of the role of circadian clocks in human cancers have shown the dysregulation or polymorphism of many circadian genes. For instance, in ovarian, prostate, colorectal and hepatic cancers, as well as gliomas and leukemia, abnormal expression of PER1, PER2, PER3, CRY1, CRY2, BMAL1, CLOCK and NPAS2 are commonly seen [11,12,13,14]. Recently, the rapid development of bioinformatics analysis techniques has facilitated the large-scale prediction of rhythm genes that are closely related to cancer development. In total, 563 circadian genes were differently expressed in hepatocellular carcinoma; three of them—CSNK1D, CSNK1E and NPAS2—had a high correlation with the overall survival rate in LIHC patients [7]. In total, 553 circadian genes with variable expressions in kidney renal clear cell carcinoma were found, and four of them—CSNK1E, GNA11, KLF9 and THRAP3—were significantly associated with the overall survival rate of KIRC patients [8]. However, differentially expressed rhythm genes regarding pancreatic cancer samples have not yet been identified. In the current investigation, we found 299 circadian genes whose expression levels were significantly altered in pancreatic cancer samples compared to normal samples. Four genes—MBOAT2, CDA, LPCAT2 and B4GALT5—were enriched in metabolic pathways. Survival analyses showed that PACA patients who had higher expression levels of MBOAT2/CDA/LPCAT2/B4GALT5 had shorter overall survival rates.

A membrane-bound O-acyltransferase domain on chromosomal band 2p25.1, known as MBOAT2, is mainly linked to adrenergic disorders, hypertrophic obstructive cardiomyopathy and multiple sclerosis [15,16,17]. Phosphatidylcholine, phosphatidylethanolamine and phosphatidylserine acyl chain remodeling are all processes in which MBOAT2 is involved. MBOAT2 may play a role in cellular interactions, cell division and biological processes connected to the immune system, including leukocyte activation, inflammatory reactions, and antigen processing and presentation in T cell receptor signaling. In pancreatic cancer, MBOAT2 overexpression increases CDK2 and CCNA2 expression levels, causing the cell cycle to advance from the G1 to the G2 phase [18]. The prognostic causes of multiple tumors may be related to the mRNA expression of MBOAT2. In cases of adrenocortical carcinoma, hepatocellular carcinoma, bladder urothelial carcinoma, endometrial carcinoma, pheochromocytoma and uveal melanoma, high mRNA levels of MBOAT2 can make a poor prognosis more likely [19]. In the literature, it has been shown that circ-MBOAT2 controls the growth of pancreatic cancer tumors and glutamine catabolism [20]. We found a strong correlation between higher MBOAT2 expression and poorer overall survival in PACA patients. When compared to normal tissues, PACA tissues had a considerably higher MBOAT2 mRNA level. The mRNA level of MBOAT2 was considerably higher in Patu−8988 and PNAC−1 cells than in HPDE6−C7 cells. Age, grade and MBOAT2 expression were all high-risk variables, according to a univariate Cox analysis. Multivariate Cox analysis indicated that MBOAT2 expression was independently associated with the overall survival rate in PACA patients. The AUC value of MBOAT2 was 0.697. The expression of MBOAT2 was negatively correlated with the infiltration of CD4+ T cells, and favorably correlated with the infiltration of CD8+ T cells and dendritic cells. Our results indicate the circadian gene MBOAT2 is an adverse prognostic factor and independent prognostic indicator. 

A deficiency of cytidine deaminase (CDA) might result in replication stress, and might lead to Bloom syndrome. CDA can also be utilized in treatments based on deaminase and deoxycytidine analogs, such as gemcitabine. CDA is a ubiquitous enzyme whose major function is to contribute to the recycling of free pyrimidines. The pyrimidine salvage pathway appears to serve two distinct functions: the recycling of pyrimidines for the synthesis of more nucleotides that will be incorporated into DNA and RNA, and the breakdown of pyrimidines to maintain a constant supply of carbon and nitrogen inside the cell. Interestingly, CDA is essential to both functions. CDA gene silencing can inhibit the proliferation of chronic myeloid leukemia cells and induce apoptosis. The efficacy of CDA is variable; cytarabine, for example, has a higher efficacy in the treatment of acute myeloid leukemia, and gemcitabine has a higher efficacy in the treatment of breast cancer. CDA is related to drug chemotherapy and can also be used as a method to mediate the treatment of endocrine tumors in thyroid cancer [21,22,23,24,25]. However, CDA polymorphisms are barely associated with lung cancer [26]. We discovered that shorter overall survival in patients with pancreatic cancer was substantially correlated with increased CDA expression. Compared to normal tissues, pancreatic cancer tissues had a considerably higher amount of CDA mRNA. The mRNA levels of CDA were considerably higher in Patu-8988 and PNAC-1 cells than in HPDE6-C7 cells. Age, grade and CDA expression were all high-risk variables, according to a univariate Cox analysis. Multivariate Cox analysis indicated that CDA expression was independently associated with the overall survival rate in PACA patients. The AUC value of CDA was 0.695. The expression of CDA was positively correlated with the infiltration of neutrophils and dendritic cells. Our results indicated that the circadian gene CDA is an adverse prognostic factor and independent prognostic indicator.

The structure of LPCAT2 (Lysophosphatidylcholine Acyltransferase 2) has not been determined, but the crystal structure of Thermotoga maritima PlsC, an enzyme belonging to the same gene family as LPCAT2, has been reported [27]. LPCAT2 is related to the production of lipid droplets, closely related to the transformation of phospholipid and is involved in various metabolic pathways. The methylation of LPCAT2 is associated with allergic rhinitis [28]. LPCAT2 controls the chemical resistance of colorectal cancer and may be associated with the dedifferentiation of thyroid cancer. The expression level of LPACT2 is associated with aggressive prostate cancers and is upregulated in breast and cervical cancers [29,30,31]. It has been reported that LPCAT2 is a target of miR-148a-5p and thus may be a prognostic gene for pancreatic cancer. The high expression of LPCAT2 was found to be associated with a poor prognosis in PACA patients. We found that higher LPCAT2 expression was significantly associated with a shorter overall survival rate in PACA patients. The mRNA level of LPCAT2 was significantly upregulated in pancreatic cancer tissues compared with normal tissues. The mRNA level of LPCAT2 was considerably higher in Patu-8988 and PNAC-1 cells than in HPDE6-C7 cells. Age, grade and LPCAT2 expression were high-risk factors, according to a univariate Cox analysis. Multivariate Cox analysis indicated that LPCAT2 expression was independently associated with the overall survival rate in PACA patients. The AUC value of LPCAT2 was 0.645. LPCAT2 expression was positively correlated with B cell, CD8+ T cell, neutrophil, macrophage and dendritic cell infiltration. Our results indicate that the circadian genes MBOAT2/CDA/LPCAT2/B4GALT5 are adverse prognostic factors and independent prognostic indicators. Our results indicated that the circadian gene LPCAT2 is an adverse prognostic factor and independent prognostic indicator.

B4GALT5 is considered an important protein in glucose metabolism, which can catalyze the synthesis of lactose ceramide. B4GALT5 is an enzyme, and it is associated with malignant tumors. miR-491-5p promotes the progression of acute myeloid leukemia by regulating the expression of B4GALT5 and the PI3K/AKT signaling pathway. The increased mRNA and protein levels of B4GALT5 are associated with poor prognosis for hepatocellular carcinoma, ovarian cancer, cervical cancer and other cancers [32,33,34,35]. In patients with pancreatic cancer, we discovered that increased B4GALT5 expression was substantially correlated with shorter overall survival. Compared to normal tissues, pancreatic cancer tissues had a considerably higher amount of B4GALT5 mRNA. The mRNA level of B4GALT5 was considerably higher in Patu-8988 and PNAC-1 cells than in HPDE6-C7 cells. Age, grade and B4GALT5 expression were high-risk factors, according to a univariate Cox analysis. Multivariate Cox analysis indicated that B4GALT5 expression was independently associated with the overall survival rate in PACA patients. The AUC value of B4GALT5 was 0.613. B4GALT5 expression was positively correlated with B cell, CD8+ T cell, neutrophil, macrophage and dendritic cell infiltration. Our results indicate that the circadian genes MBOAT2/CDA/LPCAT2/B4GALT5 are adverse prognostic factors and independent prognostic indicators. Our results indicate that the circadian gene B4GALT5 is an adverse prognostic factor and independent prognostic indicator.

Many anticancer medications have substantial time-of-administration effects, and their biological properties, including absorption, metabolism, distribution and elimination, correspond to the human circadian clock. As the medications enter tumor cells, the circadian rhythm of the cancer cells governs how they behave. This results in anticancer medications having varied effectiveness and adverse effects depending on when they were administered. In order to optimize the killing of cancer cells and lessen the harm and side effects of radiation therapy and chemotherapy, a unique kind of chronotherapy, known as chronotherapy of tumors, has been proposed and integrated into clinical treatments of tumors. According to human transcriptome microarray data, MBOAT2 expression peaked at CT10 and reached a trough at CT16; CDA expression levels peaked at CT7, while CDA expression reached a trough at CT16; LPCAT2 expression peaked at CT1 and reached a trough at CT16; and B4GALT5 expression peaked at CT4 and reached a trough at CT19. The peak expression of the four genes—MBOAT2, CDA, LPCAT2 and B4GALT5—is where we may use this data to improve the impact of anticancer medications on PACA patients. The peak expression of these four genes needs to be determined using specific tests. Thus, we may use these findings to enhance the anticancer impact of these four genes—MBOAT2, CDA, LPCAT2 and B4GALT5—in patients with PACA. Further cell tests will be performed to investigate the expression of these four genes as well as the mechanisms of their action against pancreatic cancer.

## 5. Conclusions

**Figure 9:**
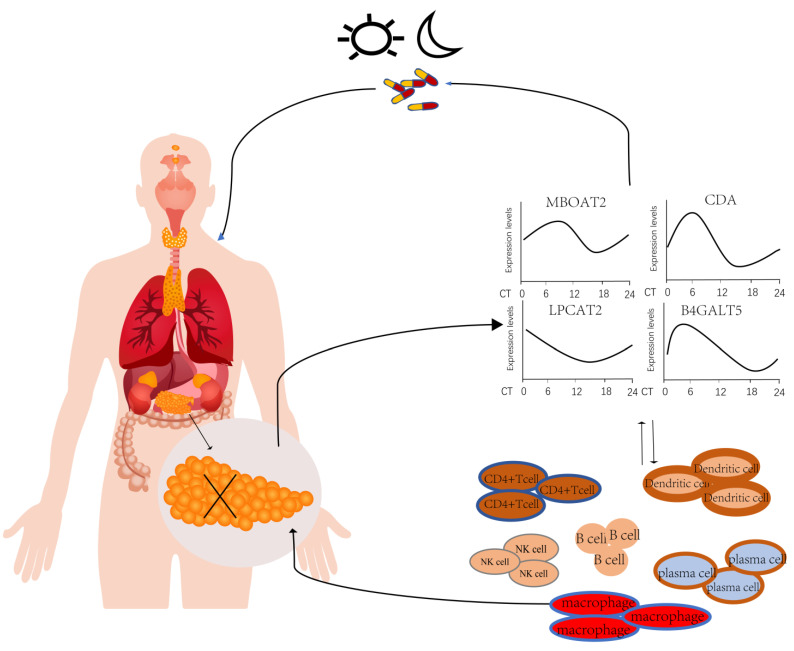
A schematic diagram of the effects and prognostic values of circadian genes MBOAT2/CDA/LPCAT2/ B4GALT5 in pancreatic cancer.

In this study, a total of 299 DERGs were identified in PACA, which were significantly abundant in the metabolic and immune response pathways. Of them, higher expression levels of MBOAT2, CDA, LPCAT2 and B4GALT5 were not only notably linked with poorer overall survival rates in PACA patients, but were also significantly related to the level of immune cell infiltration. The mRNA levels of MBOAT2, CDA, LPCAT2 and B4GALT5 were significantly higher in PACA cells than in normal human pancreatic duct epithelial cells, implying that the high expression levels of these four genes may promote the development and progression of pancreatic cancer (Figure 9). In the future, the circadian genes MBOAT2/CDA/LPCAT2/B4GALT5 may serve as prognostic and diagnostic biomarkers as well as drug targets for chronotherapy in PACA patients.

## Figures and Tables

**Table 1:** Univariate Cox analyses of the correlation between MBOAT2/CDA/LPCAT2/B4GALT5 expression and OS in PACA patients.

Parameter	Univariate Analysis		
	HR	95% CI	*p*
age	1.03	1.01−1.05	<0.05
gender	0.76	0.49−1.17	>0.05
grade	1.17	1.08−1.26	<0.005
stage	0.89	0.46−1.70	>0.05
T	1.60	0.84−3.04	>0.05
N	1.11	0.89−1.38	>0.05
MBOAT2	1.56	1.18−2.07	<0.005
CDA	1.36	1.14−1.61	<0.005
LPCAT2	1.74	1.27−2.40	<0.005
B4GALT5	1.67	1.10−2.55	<0.05

CI: confidence interval; HR: hazard ratio; T: topography; N: lymph node.

## Data Availability

In this study, data sets from the Cancer Genome Atlas database (https://portal.gdc.cancer.gov, access date: 31 May 2022), Gene Tissue Expression database (http://xena.ucsc.edu/, access date: 18 May 2016) and Circadian Gene Database (http://cgdb.biocuckoo.org, access date: 31 October 2020) were evaluated. The authors confirm that the supporting data for this study’s findings are included in the article.

## Supplementary Materials

The following supporting information can be downloaded at https://www.mdpi.com/article/10.3390/life13051116/s1, Figure S1. The survival curves of DERGs generated by the online tool KM plotter. ((A) MBOAT2; (B) CDA; (C) LPCAT2; and (D) B4GALT5.); Figure S2. The expression levels of DERGs in normal and PACA samples using the GEPIA online tool. ((A) MBOAT2; (B) CDA; (C) LPCAT2; and (D) B4GALT5); Figure S3. ROC curves of DERGs in PACA patients. ((A) MBOAT2; (B) CDA; (C) LPCAT2; and (D) B4GALT5); Figure S4. The correlation between DERGs and immune cell infiltration in PACA. (Scatter plot showing the correlation of immune cells and (A) MBOAT2; (B) CDA; (C) LPCAT2; (D) B4GALT5); Table S1. The 299 DERGs between PACA samples and non-tumor samples. Table S2. The expression levels of MBOAT2/CDA/LPCAT2/ B4GALT5 in PACA samples and non-tumor samples; Table S3. The clinical data of PACA patients.

## References

[1] Ansari D., Tingstedt B., Andersson B., Holmquist F., Sturesson C., Williamsson C., Sasor A., Borg D., Bauden M., Andersson R. (2016). Pancreatic cancer: Yesterday, today and tomorrow. Future Oncol..

[2] Lin R., Bao X., Wang H., Zhu S., Liu Z., Chen Q., Ai K., Shi B. (2021). TRPM2 promotes pancreatic cancer by PKC/MAPK pathway. Cell Death Dis..

[3] Wang S., Zheng Y., Yang F., Zhu L., Zhu X.Q., Wang Z.F., Wu X.L., Zhou C.H., Yan J.Y., Hu B.Y. (2021). The molecular biology of pancreatic adenocarcinoma: Translational challenges and clinical perspectives. Signal Transduct. Target. Ther..

[4] Vincent A., Herman J., Schulick R., Hruban R.H., Goggins M. (2011). Pancreatic cancer. Lancet.

[5] Roenneberg T., Merrow M. (2016). The Circadian Clock and Human Health. Curr. Biol..

[6] Wang X., Yang G., Wang Q., Zhao Y., Ding K., Ji C., Shi Z., Li H., Li Y., Li S. (2022). C1R, CCL2, and TNFRSF1A Genes in Coronavirus Disease-COVID-19 Pathway Serve as Novel Molecular Biomarkers of GBM Prognosis and Immune Infiltration. Dis. Markers.

[7] Liu H., Gao Y., Hu S., Fan Z., Wang X., Li S. (2021). Bioinformatics Analysis of Differentially Expressed Rhythm Genes in Liver Hepatocellular Carcinoma. Front. Genet..

[8] Li S., Wang X., Wang Q., Ding K., Chen X., Zhao Y., Gao Y., Wang Y. (2022). Effects and Prognostic Values of Circadian Genes CSNK1E/GNA11/KLF9/THRAP3 in Kidney Renal Clear Cell Carcinoma via a Comprehensive Analysis. Bioengineering.

[9] Carbone A., De Santis E., Cela O., Giambra V., Miele L., Marrone G., Grieco A., Buschbeck M., Capitanio N., Mazza T. (2021). The Histone Variant MacroH2A1 Impacts Circadian Gene Expression and Cell Phenotype in an In Vitro Model of Hepatocellular Carcinoma. Biomedicines.

[10] Emura T., Matsui S., Chen H.Y. (2019). compound.Cox: Univariate feature selection and compound covariate for predicting survival. Comput. Methods Programs Biomed..

[11] Sancar A., Van Gelder R.N. (2021). Clocks, cancer, and chronochemotherapy. Science.

[12] Winter S.L., Bosnoyan-Collins L., Pinnaduwage D., Andrulis I.L. (2007). Expression of the circadian clock genes Per1 and Per2 in sporadic and familial breast tumors. Neoplasia.

[13] Yeh K.T., Yang M.Y., Liu T.C., Chen J.C., Chan W.L., Lin S.F., Chang J.G. (2005). Abnormal expression of period 1 (PER1) in endometrial carcinoma. J. Pathol..

[14] Qu M., Zhang G., Qu H., Vu A., Wu R., Tsukamoto H., Jia Z., Huang W., Lenz H.J., Rich J.N. (2023). Circadian regulator BMAL1::CLOCK promotes cell proliferation in hepatocellular carcinoma by controlling apoptosis and cell cycle. Proc. Natl. Acad. Sci. USA.

[15] Lee D.K., Long N.P., Jung J., Kim T.J., Na E., Kang Y.P., Kwon S.W., Jang J. (2019). Integrative lipidomic and transcriptomic analysis of X-linked adrenoleukodystrophy reveals distinct lipidome signatures between adrenomyeloneuropathy and childhood cerebral adrenoleukodystrophy. Biochem. Biophys. Res. Commun..

[16] Sonnenschein K., Wilczek A.L., de Gonzalo-Calvo D., Pfanne A., Derda A.A., Zwadlo C., Bavendiek U., Bauersachs J., Fiedler J., Thum T. (2019). Serum circular RNAs act as blood-based biomarkers for hypertrophic obstructive cardiomyopathy. Sci. Rep..

[17] Schmied M.C., Zehetmayer S., Reindl M., Ehling R., Bajer-Kornek B., Leutmezer F., Zebenholzer K., Hotzy C., Lichtner P., Meitinger T. (2012). Replication study of multiple sclerosis (MS) susceptibility alleles and correlation of DNA-variants with disease features in a cohort of Austrian MS patients. Neurogenetics.

[18] Li Z., Zhuang H., Chen X., Zhang Y., Ma Z., Wang S., Yan Q., Zhou Z., Huang S., Zhang C. (2022). Identification of MBOAT2 as an Unfavorable Biomarker Correlated with KRAS Activation and Reduced CD8(+) T-Cell Infiltration in Pancreatic Cancer. J. Oncol..

[19] Xie L.Y., Huang H.Y., Fang T., Liang J.Y., Hao Y.L., Zhang X.J., Xie Y.X., Wang C., Tan Y.H., Zeng L. (2022). A Prognostic Survival Model of Pancreatic Adenocarcinoma Based on Metabolism-Related Gene Expression. Front. Genet..

[20] Zhou X., Liu K., Cui J., Xiong J., Wu H., Peng T., Guo Y. (2021). Circ-MBOAT2 knockdown represses tumor progression and glutamine catabolism by miR-433-3p/GOT1 axis in pancreatic cancer. J. Exp. Clin. Cancer Res..

[21] Frances A., Cordelier P. (2020). The Emerging Role of Cytidine Deaminase in Human Diseases: A New Opportunity for Therapy?. Mol. Ther..

[22] Wei X.F., Feng Y.F., Chen Q.L., Zhang Q.K. (2018). CDA gene silencing regulated the proliferation and apoptosis of chronic myeloid leukemia K562 cells. Cancer Cell Int..

[23] Abraham A., Varatharajan S., Abbas S., Zhang W., Shaji R.V., Ahmed R., Abraham A., George B., Srivastava A., Chandy M. (2012). Cytidine deaminase genetic variants influence RNA expression and cytarabine cytotoxicity in acute myeloid leukemia. Pharmacogenomics.

[24] Lam S.W., van der Noort V., van der Straaten T., Honkoop A.H., Peters G.J., Guchelaar H.J., Boven E. (2018). Single-nucleotide polymorphisms in the genes of CES2, CDA and enzymatic activity of CDA for prediction of the efficacy of capecitabine-containing chemotherapy in patients with metastatic breast cancer. Pharmacol. Res..

[25] Yuan M.H., Wei L.X., Zhou R.S., Xu H.F., Wang J.Y., Bai Q.R. (2017). Therapeutic effects of adenovirus-mediated CD and NIS expression combined with Na(131)I/5-FC on human thyroid cancer. Oncol. Lett..

[26] Zhou M., Wan H.Y., Gao B.L., Ding Y.J., Jun R.X. (2012). Genetic polymorphisms of XPD and CDA and lung cancer risk. Oncol. Lett..

[27] Hamano F., Matoba K., Hashidate-Yoshida T., Suzuki T., Miura K., Hishikawa D., Harayama T., Yuki K., Kita Y., Noda N.N. (2021). Mutagenesis and homology modeling reveal a predicted pocket of lysophosphatidylcholine acyltransferase 2 to catch Acyl-CoA. FASEB J..

[28] Abate W., Alrammah H., Kiernan M., Tonks A.J., Jackson S.K. (2020). Lysophosphatidylcholine acyltransferase 2 (LPCAT2) co-localises with TLR4 and regulates macrophage inflammatory gene expression in response to LPS. Sci. Rep..

[29] Agarwal A.K., Garg A. (2010). Enzymatic activity of the human 1-acylglycerol-3-phosphate-O-acyltransferase isoform 11: Upregulated in breast and cervical cancers. J. Lipid Res..

[30] Cotte A.K., Aires V., Ghiringhelli F., Delmas D. (2018). LPCAT2 controls chemoresistance in colorectal cancer. Mol. Cell. Oncol..

[31] Ma B., Jiang H., Wen D., Hu J., Han L., Liu W., Xu W., Shi X., Wei W., Liao T. (2019). Transcriptome Analyses Identify a Metabolic Gene Signature Indicative of Dedifferentiation of Papillary Thyroid Cancer. J. Clin. Endocrinol. Metab..

[32] Wu Y., Zhao B., Chen X., Geng X., Zhang Z. (2022). Circ_0009910 sponges miR-491-5p to promote acute myeloid leukemia progression through modulating B4GALT5 expression and PI3K/AKT signaling pathway. Int. J. Lab. Hematol..

[33] Han Y., Li Z., Wu Q., Liu H., Sun Z., Wu Y., Luo J. (2022). B4GALT5 high expression associated with poor prognosis of hepatocellular carcinoma. BMC Cancer.

[34] Xu X., Wu Y., Jia G., Zhu Q., Li D., Xie K. (2023). A signature based on glycosyltransferase genes provides a promising tool for the prediction of prognosis and immunotherapy responsiveness in ovarian cancer. J. Ovarian Res..

[35] Narayan G., Murty V.V. (2010). Integrative genomic approaches in cervical cancer: Implications for molecular pathogenesis. Future Oncol..

