# Supplementary material for: Circadian Genes MBOAT2/CDA/LPCAT2/B4GALT5 in the Metabolic Pathway Serve as New Biomarkers of PACA Prognosis and Immune Infiltration
Source: Life (Basel). 2023 Apr 30;13(5):1116. doi: 10.3390/life13051116 (PMC10221058; doi:10.3390/life13051116)
Supplement: Supplementary file 1 [file life-13-01116-s001.zip › Table S1.pdf]

Table S1. The 299 DERGs between PACA samples and non-tumor samples.

gene	ID	conMean	treatMean	logFC	P	P.adjust
CTRL	1506	8.708854	0.305244	-8.40361	8.11E-55	1.45E-53
CBS	875	5.750973	0.09084	-5.66013	3.13E-54	4.07E-53
PLIN5	440503	5.522534	0.714391	-4.80814	1.12E-53	1.06E-52
ECHDC2	55268	6.68329	2.599627	-4.08366	1.55E-53	1.37E-52
GATM	2628	8.229525	4.179662	-4.04986	3.73E-48	1.24E-47
MKNK1	8569	6.318557	2.289096	-4.02946	2.02E-55	5.02E-54
RBM4	5936	5.279773	1.82848	-3.45129	1.79E-54	2.67E-53
P2RX1	5023	4.515311	1.121315	-3.394	7.2E-51	3.18E-50
RIC3	79608	3.828666	0.675969	-3.1527	8.47E-55	1.5E-53
NUCB2	4925	6.1141	3.090183	-3.02392	4.94E-50	1.96E-49
TRIB1	4672	1.594825	2.639481	1.044656	1.59E-31	2.81E-31
PABPC1L	80336	5.143831	2.403674	-2.74016	1.19E-53	1.12E-52
RPL14	9045	8.638768	5.902134	-2.73663	1.56E-53	1.38E-52
GTF2IRD2	84163	3.234117	0.51124	-2.72288	3.29E-53	2.5E-52
SH3YL1	26751	5.566077	2.876393	-2.68968	2.41E-53	1.94E-52
RAB24	53917	5.119091	2.464659	-2.65443	2.64E-54	3.57E-53
CA4	762	3.502619	0.960615	-2.542	3.71E-48	1.23E-47
ARHGEF4	50649	3.695867	1.165347	-2.53052	2.98E-51	1.4E-50
TCEA3	6920	6.158054	3.695025	-2.46303	7.32E-49	2.57E-48
EPHX2	2053	4.651584	2.261008	-2.39058	1.02E-49	3.91E-49
RGL3	57139	4.825072	2.46196	-2.36311	1.58E-52	9.65E-52
SLC31A2	1318	2.939072	0.675021	-2.26405	6.17E-55	1.17E-53
MTMR3	8897	3.160676	0.900197	-2.26048	1.07E-54	1.81E-53
RPS23	6228	9.303255	7.0477	-2.25556	1.86E-54	2.77E-53
FAM153B	202134	2.386934	0.165164	-2.22177	5.23E-54	5.99E-53
PHGDH	26227	4.876062	2.676637	-2.19942	2.04E-47	6.36E-47
RGPD5	84220	2.193662	0.002506	-2.19116	3.96E-57	2.91E-55
NAIP	4671	2.191511	0.09675	-2.09476	2.73E-55	6.31E-54
UBA5	79876	4.583617	2.555938	-2.02768	1.34E-53	1.22E-52
ATF7IP2	80063	3.551392	1.524328	-2.02706	1.2E-52	7.56E-52
CYHR1	50626	4.894421	2.876365	-2.01806	3.35E-53	2.53E-52
LRRC37A2	474170	2.584426	0.566436	-2.01799	1.17E-53	1.11E-52
NPAS2	4862	4.329349	2.340525	-1.98882	2.93E-51	1.38E-50
NQO2	4835	3.703809	1.716587	-1.98722	8.77E-53	5.75E-52
MTHFS	10588	4.00619	2.080674	-1.92552	1.88E-52	1.13E-51
HAAO	23498	3.813033	1.902848	-1.91019	1.08E-53	1.04E-52
TRAF5	7188	4.031615	2.147591	-1.88402	1.92E-52	1.15E-51
CDH23	64072	2.316	0.472368	-1.84363	7.05E-55	1.31E-53
FAM153A	285596	1.965045	0.124356	-1.84069	3.03E-54	3.97E-53
ANKRD36	375248	2.199317	0.382527	-1.81679	2.41E-54	3.33E-53
MRRF	92399	3.653321	1.858096	-1.79523	2.33E-53	1.89E-52
METTL3	56339	4.301945	2.512016	-1.78993	4.66E-54	5.48E-53

GCSH	2653	3.139522	1.386299	-1.75322	9.36E-52	4.81E-51
ELP2	55250	4.303275	2.553195	-1.75008	2.49E-54	3.41E-53
RALGAPA1	253959	2.887902	1.13866	-1.74924	1.68E-53	1.46E-52
RPL22	6146	7.450471	5.723401	-1.72707	5.43E-54	6.17E-53
RPL21	6144	8.584119	6.860139	-1.72398	1.1E-50	4.76E-50
SLC25A36	55186	4.526554	2.806415	-1.72014	1.83E-53	1.56E-52
RCL1	10171	3.5039	1.786245	-1.71766	1.64E-49	6.15E-49
LDHB	3945	7.066992	5.380179	-1.68681	2.11E-50	8.78E-50
ULK1	8408	4.931546	3.250134	-1.68141	2.88E-50	1.18E-49
GGA2	23062	5.198467	3.520635	-1.67783	4.58E-54	5.41E-53
KDM4C	23081	3.053289	1.398882	-1.65441	5.29E-53	3.73E-52
ECHDC3	79746	3.440627	1.802349	-1.63828	3.02E-46	8.75E-46
SMARCE1	6605	4.29256	2.658968	-1.63359	1.33E-54	2.12E-53
GIMAP5	55340	1.980868	0.350952	-1.62992	1.7E-54	2.56E-53
SFXN2	118980	2.666599	1.060545	-1.60605	9.36E-52	4.81E-51
TYK2	7297	5.366595	3.766377	-1.60022	4.81E-55	9.64E-54
SEC63	11231	5.244271	3.673097	-1.57117	5.67E-52	3.03E-51
ZNF577	84765	2.628973	1.071029	-1.55794	8.61E-55	1.52E-53
FAM162A	26355	5.347925	3.792967	-1.55496	6.77E-49	2.39E-48
EIF3E	3646	6.973837	5.423927	-1.54991	3.54E-53	2.65E-52
GOLGA6L9	440295	2.018161	0.484605	-1.53356	3.7E-54	4.58E-53
MXD3	83463	2.838117	1.307016	-1.5311	7.2E-51	3.18E-50
ZMAT1	84460	2.895974	1.382342	-1.51363	1.9E-48	6.43E-48
TSEN2	80746	2.806123	1.30171	-1.50441	5.15E-52	2.78E-51
LRIG1	26018	5.066168	3.562544	-1.50362	7.44E-45	1.99E-44
MDM2	4193	3.913624	2.416354	-1.49727	1.73E-51	8.47E-51
ASNS	440	4.400614	2.905254	-1.49536	8.95E-44	2.28E-43
DDHD2	23259	3.712155	2.234757	-1.4774	1.38E-52	8.6E-52
KANK1	23189	3.409649	1.936686	-1.47296	1.14E-50	4.92E-50
ALG13	79868	3.62291	2.154058	-1.46885	3.94E-53	2.91E-52
CARD8	22900	3.160832	1.700962	-1.45987	7.29E-55	1.33E-53
NR2C1	7181	3.51889	2.060955	-1.45794	1.87E-53	1.59E-52
CCDC85C	317762	2.740539	1.2954	-1.44514	2.75E-51	1.3E-50
CXCL2	2920	4.25667	2.812996	-1.44367	3.9E-13	5.05E-13
FLCN	201163	3.457535	2.026246	-1.43129	5.5E-54	6.19E-53
ATP9B	374868	2.604838	1.213804	-1.39103	9.01E-54	9.04E-53
CLYBL	171425	2.354577	0.980303	-1.37427	5.78E-55	1.12E-53
NPM1	4869	8.121727	6.771076	-1.35065	6.87E-52	3.62E-51
PRR4	11272	1.751938	0.413584	-1.33835	5.27E-50	2.09E-49
FHIT	2272	2.651292	1.315451	-1.33584	9.16E-46	2.57E-45
GON4L	54856	3.468198	2.138068	-1.33013	3.81E-53	2.82E-52
CSNK1D	1453	5.317582	3.990689	-1.32689	4.02E-54	4.9E-53
TBC1D3B	414059	1.363325	0.038418	-1.32491	1.25E-54	2.03E-53
TRA2B	6434	4.610626	3.300829	-1.3098	2.37E-54	3.29E-53

PDPK1	5170	3.158151	1.857233	-1.30092	3.52E-53	2.64E-52
ANAPC4	29945	3.422317	2.130464	-1.29185	2.07E-54	3E-53
TTC21A	199223	2.146607	0.854903	-1.2917	8.92E-53	5.83E-52
ANK3	288	2.236973	0.948741	-1.28823	5.57E-51	2.5E-50
TOMM7	54543	7.573445	6.285771	-1.28767	1.6E-48	5.45E-48
SNURF	8926	1.582508	0.304758	-1.27775	1.04E-53	1.01E-52
SLC37A3	84255	3.592471	2.315672	-1.2768	6.54E-51	2.9E-50
KREMEN1	83999	1.276083	0	-1.27608	5.14E-65	2.54E-62
TC2N	123036	5.042326	3.768972	-1.27335	3.7E-40	8.16E-40
DDX42	11325	5.282593	4.019259	-1.26333	1.57E-53	1.38E-52
C11orf58	10944	5.003328	3.742991	-1.26034	1.34E-53	1.22E-52
PASK	23178	2.440157	1.184967	-1.25519	2.98E-51	1.4E-50
GSTM4	2948	3.598758	2.346071	-1.25269	1.36E-44	3.6E-44
RAB3D	9545	3.977903	2.740489	-1.23741	9.52E-40	2.08E-39
ARNTL	406	2.718151	1.486743	-1.23141	7.3E-47	2.19E-46
ADCY4	196883	2.73971	1.514663	-1.22505	9.61E-50	3.69E-49
HPCAL4	51440	1.384279	0.164447	-1.21983	4.95E-44	1.27E-43
HMGB1	3146	5.973418	4.754812	-1.21861	1.55E-54	2.4E-53
FABP5	2171	4.076527	2.870316	-1.20621	3.69E-38	7.66E-38
CRAT	1384	5.267714	4.065326	-1.20239	2.86E-41	6.56E-41
TTLL4	9654	3.271933	2.091513	-1.18042	4.11E-52	2.26E-51
RAB43	339122	1.74495	0.564784	-1.18017	1.92E-53	1.62E-52
FKBP5	2289	4.80624	3.656038	-1.1502	1.29E-18	1.82E-18
OGG1	4968	2.652832	1.510519	-1.14231	1.38E-53	1.25E-52
DNAJC10	54431	4.180022	3.038952	-1.14107	8E-47	2.4E-46
TCERG1	10915	3.887814	2.748482	-1.13933	8.36E-52	4.34E-51
DCAF4	26094	2.931437	1.795811	-1.13563	5.56E-53	3.89E-52
BEND7	222389	2.757264	1.629685	-1.12758	6.09E-51	2.72E-50
ANAPC1	64682	2.207878	1.083321	-1.12456	3.24E-53	2.46E-52
HPS4	89781	3.430998	2.312151	-1.11885	5.51E-53	3.87E-52
CSNK1E	1454	5.460011	4.34633	-1.11368	1.68E-50	7.07E-50
DNAJC19	131118	3.817013	2.706435	-1.11058	2.84E-51	1.34E-50
MSL1	339287	4.839747	3.751531	-1.08822	6.67E-52	3.52E-51
CYP4V2	285440	3.363599	2.27601	-1.08759	3.09E-49	1.12E-48
NOL8	55035	3.340887	2.2606	-1.08029	6.81E-54	7.32E-53
BCL11A	53335	1.754229	0.68335	-1.07088	4.23E-46	1.21E-45
THEM	117145	3.222989	2.160731	-1.06226	2.9E-50	1.19E-49
THEM4	117145	3.222989	2.160731	-1.06226	2.9E-50	1.19E-49
UBE2B	7320	5.493971	4.436388	-1.05758	1.44E-52	8.92E-52
HOOK1	51361	3.187095	2.130719	-1.05638	1.5E-43	3.78E-43
ANKRD18A	253650	1.510995	0.458362	-1.05263	2.76E-46	8E-46
XKR6	286046	1.402382	0.35094	-1.05144	6.25E-52	3.31E-51
TMEM25	84866	3.008324	1.9592	-1.04912	8.63E-43	2.1E-42
SNRPA1	6627	4.103907	3.06861	-1.0353	5.43E-48	1.77E-47

SNRPA2	6627	4.103907	3.06861	-1.0353	5.43E-48	1.77E-47
PLEKHG4	25894	2.280002	1.252059	-1.02794	6.35E-43	1.56E-42
COX11	1353	3.587802	2.562733	-1.02507	1.44E-48	4.95E-48
SEMA3B	7869	4.697587	3.677418	-1.02017	9.11E-25	1.42E-24
RORA	6095	2.255003	1.248906	-1.0061	1.99E-49	7.36E-49
FOXO1	2308	2.38908	3.389117	1.000036	1.18E-41	2.75E-41
DIP2B	57609	1.777146	2.78095	1.003803	1.4E-53	1.27E-52
NEDD9	4739	2.126741	3.133052	1.006311	4.84E-39	1.03E-38
ITPKB	3707	1.716023	2.722788	1.006765	1.23E-48	4.23E-48
CBL	867	0.991073	1.999641	1.008568	1.78E-51	8.72E-51
TFE3	7030	3.175106	4.184042	1.008936	1.68E-53	1.46E-52
G6PD	2539	2.803245	3.816208	1.012962	2.5E-51	1.19E-50
GNG2	54331	1.169368	2.184446	1.015078	4.6E-33	8.43E-33
RMND5A	64795	2.314931	3.339366	1.024434	3.97E-56	1.5E-54
ARHGAP26	23092	1.468222	2.494084	1.025862	1.05E-39	2.29E-39
PDCL3	79031	2.3472	3.376341	1.029141	9.08E-54	9.11E-53
ZCCHC17	51538	2.901476	3.932308	1.030832	5.06E-55	1E-53
CPPED1	55313	1.193133	2.226718	1.033585	5.45E-47	1.65E-46
CD200	4345	1.594825	2.639481	1.044656	1.59E-31	2.81E-31
MAPKAPK2	9261	4.354926	5.399707	1.044782	2.34E-50	9.69E-50
TRANK1	9881	1.130232	2.179094	1.048861	2.09E-48	7.03E-48
TRANK2	9881	1.130232	2.179094	1.048861	2.09E-48	7.03E-48
PYGL	5836	2.009358	3.059697	1.050338	1.19E-33	2.21E-33
MIDN	90007	3.990917	5.042907	1.05199	1.69E-34	3.21E-34
AGMAT	79814	0.33879	1.39495	1.05616	4.24E-51	1.95E-50
TRAFD1	10906	2.61169	3.667998	1.056308	1.65E-53	1.44E-52
PLOD1	5351	4.006264	5.073037	1.066772	3.76E-42	8.94E-42
TMEM127	55654	3.524485	4.595447	1.070962	4.17E-53	3.05E-52
TREM1	54210	0.467623	1.539517	1.071894	1.45E-33	2.68E-33
IL23A	51561	0.601654	1.682755	1.081101	6.66E-46	1.89E-45
GSK3B	2932	2.029602	3.121141	1.091539	1.05E-55	3.07E-54
AQP9	366	0.371123	1.466169	1.095046	4.15E-35	8.01E-35
GIMAP7	168537	1.999536	3.099337	1.0998	5.17E-24	7.95E-24
RNF24	11237	1.440595	2.559248	1.118653	3.51E-45	9.53E-45
ABLIM1	3983	3.252575	4.371322	1.118747	1.53E-45	4.25E-45
SP1	6667	2.949161	4.075188	1.126026	6.25E-52	3.31E-51
FCGR2A	2212	1.978869	3.106088	1.127219	3.55E-26	5.67E-26
SWAP70	23075	2.243945	3.374093	1.130148	3.92E-46	1.12E-45
SIPA1L2	57568	1.470723	2.606449	1.135726	4.02E-44	1.04E-43
FBXO28	23219	1.852195	3.001625	1.14943	2.1E-56	9.33E-55
ITGA5	3678	3.307026	4.460767	1.153741	2.71E-31	4.78E-31
MPZL1	9019	3.600486	4.760081	1.159594	3.17E-48	1.06E-47
SUSD3	203328	0.578919	1.740159	1.16124	2.81E-44	7.31E-44
CHMP1A	5119	4.218369	5.386868	1.168499	4.36E-56	1.58E-54

ATP10A	57194	0.471116	1.65809	1.186974	1.29E-51	6.48E-51
ZNF629	23361	1.707514	2.898507	1.190993	5.33E-56	1.82E-54
GPA33	10223	0.07168	1.265734	1.194054	3.99E-49	1.44E-48
HPSE	10855	0.335969	1.533628	1.19766	6.17E-55	1.17E-53
ITGA6	3655	4.159078	5.357051	1.197974	7.81E-35	1.5E-34
MCL1	4170	5.860543	7.06113	1.200586	1.68E-38	3.52E-38
CSF2RA	1438	0.754824	1.963828	1.209004	1.64E-38	3.45E-38
TMEM140	55281	2.367818	3.580043	1.212225	3.79E-49	1.37E-48
NFAM1	150372	0.501542	1.719598	1.218056	6E-48	1.95E-47
FOS	2353	5.834716	7.055883	1.221168	5.25E-12	6.69E-12
KIF3C	3797	0.78063	2.002656	1.222026	7.29E-50	2.84E-49
SYTL3	94120	0.646763	1.885898	1.239135	2.66E-50	1.09E-49
RARA	5914	2.581822	3.823727	1.241904	6.79E-50	2.65E-49
INHBB	3625	1.226199	2.477821	1.251623	1.08E-36	2.17E-36
PTPRJ	5795	1.789782	3.060491	1.270709	3.33E-55	7.34E-54
DOK4	55715	2.905551	4.189161	1.28361	4.76E-49	1.7E-48
CPNE7	27132	0.772701	2.061138	1.288437	9.18E-41	2.07E-40
ST6GAL1	6480	1.783536	3.07995	1.296414	4.51E-39	9.65E-39
PRF1	5551	0.551595	1.856118	1.304524	1.9E-51	9.24E-51
LPCAT2	54947	1.325749	2.641642	1.315893	3.95E-47	1.21E-46
PCYOX1	51449	2.609632	3.927822	1.31819	3.21E-54	4.14E-53
SECTM1	6398	1.384251	2.709964	1.325713	5.08E-36	1E-35
VSIG4	11326	2.424449	3.751388	1.326939	1.52E-21	2.25E-21
OSGIN2	734	1.347719	2.678172	1.330454	2.55E-55	5.96E-54
TSPAN18	90139	0.776087	2.118201	1.342113	6.87E-49	2.42E-48
SNN	8303	1.832974	3.194239	1.361265	5.49E-52	2.95E-51
RUNX2	860	0.422974	1.807063	1.384089	1.45E-50	6.16E-50
STMN3	50861	2.813994	4.199428	1.385434	6.41E-40	1.41E-39
MSRB2	22921	2.402017	3.82489	1.422873	2.29E-54	3.21E-53
RSAD2	91543	0.87861	2.303636	1.425026	1.33E-44	3.52E-44
UBTD1	80019	2.205218	3.637573	1.432355	8.49E-53	5.59E-52
TNFSF10	8743	3.229816	4.673501	1.443685	2.37E-45	6.51E-45
PJA1	64219	2.392864	3.842355	1.449491	2.51E-55	5.87E-54
ABHD2	11057	3.768483	5.219875	1.451392	8.26E-43	2.02E-42
FOXJ1	2302	0.337325	1.792783	1.455457	4.8E-37	9.7E-37
NYNRIN	57523	1.384995	2.843605	1.45861	9.36E-53	6.08E-52
DTX3L	151636	2.613174	4.07669	1.463516	1.57E-51	7.76E-51
LDLR	3949	3.0679	4.54541	1.477509	8.16E-31	1.42E-30
CD300LF	146722	0.394983	1.872629	1.477646	4.83E-48	1.58E-47
LITAF	9516	4.010559	5.502443	1.491884	4.22E-46	1.21E-45
MYO7B	4648	0.574497	2.076238	1.501742	8.65E-30	1.48E-29
CANT1	124583	3.120411	4.626203	1.505792	2.48E-57	2.08E-55
NCF4	4689	1.423893	2.929854	1.505962	4.61E-42	1.09E-41
CPD	1362	2.436071	3.947217	1.511146	5.95E-56	1.97E-54

TRIB1	10221	2.803025	4.318768	1.515743	7.44E-45	1.99E-44
SLC2A3	6515	1.981474	3.500685	1.519211	4.54E-33	8.32E-33
WAS	7454	1.236677	2.759781	1.523104	3.16E-42	7.54E-42
TMCC3	57458	0.772479	2.30322	1.530741	9.78E-54	9.65E-53
FCGBP	8857	1.44606	2.987222	1.541161	2.34E-24	3.62E-24
EID1	23741	4.508653	6.059492	1.550838	3.49E-52	1.96E-51
ISM1	140862	1.238933	2.794096	1.555162	1E-31	1.79E-31
HKDC1	80201	1.871581	3.429778	1.558196	2.56E-38	5.34E-38
DBN1	1627	2.980444	4.544391	1.563947	1.68E-48	5.71E-48
CHIC2	26511	2.260202	3.845879	1.585676	4.14E-55	8.6E-54
C1orf54	79630	1.51463	3.110999	1.596369	6.56E-49	2.32E-48
CD3E	916	0.964288	2.564441	1.600152	3.6E-42	8.57E-42
PPT1	5538	3.73459	5.338283	1.603692	8.85E-52	4.57E-51
CORO1C	23603	2.689788	4.312506	1.622718	9.91E-51	4.3E-50
ABCG1	9619	1.306629	2.930938	1.624309	2.89E-54	3.83E-53
TSPAN2	10100	0.831586	2.45792	1.626334	5.23E-46	1.49E-45
RAB20	55647	3.298862	4.938322	1.63946	2.68E-53	2.11E-52
DUSP1	1843	5.443167	7.08529	1.642123	3.46E-25	5.43E-25
COTL1	23406	3.40368	5.046246	1.642566	6.46E-46	1.83E-45
KIF11	3832	0.345188	1.995871	1.650682	2.65E-56	1.1E-54
VMO1	284013	0.682967	2.336365	1.653399	1.15E-53	1.1E-52
PFKFB3	5209	2.82851	4.494614	1.666104	7.01E-45	1.87E-44
PLEK	5341	0.849172	2.521259	1.672088	1.29E-44	3.42E-44
CRISPLD2	83716	2.887528	4.579562	1.692034	7.3E-38	1.5E-37
TP53INP2	58476	1.913734	3.606615	1.69288	2.49E-53	1.99E-52
IFIT2	3433	0.870269	2.614411	1.744142	5.58E-52	2.99E-51
HCK	3055	1.20535	2.950657	1.745307	3.16E-45	8.6E-45
ARID5B	84159	1.205287	2.959643	1.754355	1.53E-52	9.38E-52
CD83	9308	0.904888	2.680037	1.775148	7.12E-50	2.78E-49
DACT1	51339	0.910874	2.704769	1.793895	4.61E-48	1.51E-47
GNS	2799	3.308168	5.10612	1.797952	3.17E-55	7.06E-54
CHSY1	22856	1.717547	3.538022	1.820475	3.08E-54	4.02E-53
EMP1	2012	3.092388	4.916896	1.824508	2.74E-41	6.29E-41
LMNB1	4001	1.226242	3.087719	1.861476	2.12E-55	5.21E-54
ALOX5AP	241	2.571182	4.488757	1.917575	1.32E-38	2.77E-38
BIRC3	330	2.020205	3.955969	1.935764	2.03E-39	4.4E-39
PIK3IP1	113791	2.434452	4.377775	1.943322	6.33E-53	4.34E-52
MBOAT2	129642	1.104711	3.078393	1.973681	3.81E-55	8.03E-54
CYP27A1	1593	2.180557	4.182765	2.002208	7.45E-53	4.99E-52
HAPLN3	145864	0.969841	3.014763	2.044922	8.38E-47	2.51E-46
TIMP2	7077	4.866313	6.919698	2.053385	4.79E-47	1.46E-46
PTGS2	5743	0.295263	2.351243	2.05598	2.16E-51	1.04E-50
MIA	8190	0.643203	2.706804	2.063601	8.21E-31	1.43E-30
CD93	22918	1.927482	4.013907	2.086425	8.22E-53	5.44E-52

IL13RA1	3597	3.071649	5.17554	2.103891	2.79E-56	1.13E-54
CDC42EP2	10435	1.614641	3.793763	2.179122	2.45E-54	3.37E-53
CTSD	1509	6.972443	9.181294	2.208851	5.48E-54	6.19E-53
JUNB	3726	5.428323	7.640544	2.212221	3.58E-39	7.69E-39
IFIT3	3437	1.620694	3.850477	2.229783	1.29E-51	6.48E-51
KRT23	25984	1.045053	3.295575	2.250523	2.1E-36	4.17E-36
RCN3	57333	2.688342	4.942736	2.254394	3.21E-49	1.16E-48
MYL9	10398	4.864455	7.119677	2.255222	5.82E-44	1.5E-43
SOCS3	9021	3.238565	5.532863	2.294298	1.27E-42	3.08E-42
RNASE6	6039	1.609025	3.91641	2.307385	6.19E-48	2.01E-47
TNFAIP6	7130	0.290232	2.611447	2.321215	2.53E-51	1.2E-50
CYBRD1	79901	2.587138	4.952255	2.365117	1.95E-47	6.08E-47
SPON1	10418	1.624285	4.02548	2.401195	5.37E-47	1.63E-46
OASL	8638	0.304625	2.796489	2.491864	3.5E-51	1.63E-50
ATP1B1	481	5.196712	7.692027	2.495315	2.42E-52	1.41E-51
FSTL1	11167	3.189596	5.692106	2.50251	4.68E-48	1.54E-47
SLC6A6	6533	1.390401	3.993993	2.603591	1.6E-52	9.78E-52
CD248	57124	2.430075	5.051117	2.621043	2.53E-47	7.86E-47
CDR2L	30850	1.421536	4.050906	2.62937	7.17E-55	1.32E-53
B4GALT5	9334	2.298763	4.931888	2.633125	4.4E-56	1.59E-54
PMEPA1	56937	3.483695	6.242638	2.758943	2.47E-48	8.3E-48
VNN1	8876	0.650029	3.411773	2.761744	1.97E-46	5.78E-46
CTSS	1520	2.608702	5.464659	2.855957	4.68E-48	1.54E-47
PLAUR	5329	1.722467	4.593376	2.87091	9.17E-50	3.52E-49
BASP1	10409	1.220222	4.107882	2.887659	6.35E-52	3.36E-51
TMEM45B	120224	0.667099	3.755048	3.08795	7.54E-55	1.37E-53
IL1RN	3557	0.676877	3.792243	3.115367	1.89E-47	5.9E-47
CDA	978	0.607673	3.805243	3.197571	1.6E-56	7.82E-55
C1Q	712	3.626318	6.841475	3.215157	1.01E-48	3.51E-48
C1QA	702	1.777333	2.863275	1.085942	1.08E-48	3.75E-48
UBE2C	11065	0.52678	3.74195	3.215169	1.87E-55	4.77E-54
FAM3D	131177	0.477527	3.709626	3.232099	3.34E-47	1.03E-46
SERPINE1	5054	2.887172	6.442419	3.555247	7.32E-49	2.57E-48
CAMK2N1	55450	2.115206	5.82817	3.712964	5.73E-58	6.24E-56
S100A11	6282	6.421565	10.84395	4.422387	3.39E-51	1.58E-50
APOL1	8542	2.087471	6.64413	4.556659	2.36E-56	1E-54
TFF2	7032	1.126983	6.480692	5.353709	1.47E-43	3.69E-43
