# Supplementary material for: Circadian Genes MBOAT2/CDA/LPCAT2/B4GALT5 in the Metabolic Pathway Serve as New Biomarkers of PACA Prognosis and Immune Infiltration
Source: Life (Basel). 2023 Apr 30;13(5):1116. doi: 10.3390/life13051116 (PMC10221058; doi:10.3390/life13051116)
Supplement: Supplementary file 1 [file life-13-01116-s001.zip › Table S2.pdf]

Table S2.The expression levels of MBOAT2/CDA/LPCAT2/ B4GALT5 in PACA samples and non-tumor samples.

ID	MBOAT2	CDA	LPCAT2	B4GALT5	Type
GTEX-S33H-1226-SM-4AD69	0.720877	0.218443	1.50317	2.086464	Normal
GTEX-VJYA-0826-SM-4KL1M	1.385663	0.07317	1.345928	2.165812	Normal
GTEX-ZF3C-2026-SM-4WWB5	1.216851	0.425327	1.12676	2.700784	Normal
GTEX-XUW1-1726-SM-4BOOZ	0.915508	0.284174	1.428501	1.831362	Normal
GTEX-OOBJ-1026-SM-3NB2L	0.786007	0.505156	1.133805	1.960132	Normal
GTEX-ZLFU-0726-SM-57WF6	1.178757	0.356554	1.407112	2.158608	Normal
GTEX-QEL4-1326-SM-447AD	0.782345	0.670413	1.416678	1.722962	Normal
GTEX-11DXX-0926-SM-5H112	0.841368	0.388208	1.493985	1.987748	Normal
GTEX-ZAB4-1726-SM-5HL8C	0.806976	0.658086	1.412532	1.649932	Normal
GTEX-145ME-0326-SM-5S2QO	1.470905	0.541689	1.46238	1.957646	Normal
GTEX-YB5E-0526-SM-4VDSD	1.235201	1.047028	0.938324	2.706037	Normal
GTEX-12WSG-1026-SM-5EGII	1.13285	0.269995	1.501442	2.172487	Normal
GTEX-ZV7C-0726-SM-59HKH	0.977754	0.49302	1.591708	2.687454	Normal
GTEX-V1D1-0726-SM-4JBH7	1.286225	0.861834	1.557051	2.30673	Normal
GTEX-TKQ2-0426-SM-4DXUO	1.072054	0.185231	1.449766	2.163175	Normal
GTEX-RWS6-1126-SM-47JXC	1.261035	0.28292	0.947897	1.836539	Normal
GTEX-145MO-2126-SM-5Q5CZ	0.952509	0.507929	1.121074	2.021858	Normal
GTEX-13W3W-1026-SM-5IFG4	0.825062	0.834084	1.414598	1.852153	Normal
GTEX-N7MT-1626-SM-3LK71	0.790402	0.622324	1.264945	2.332448	Normal
GTEX-131XG-1426-SM-5GCMO	0.958688	0.694024	1.369111	2.220906	Normal
GTEX-11XUK-0626-SM-5N9ES	0.914684	0.596816	0.984849	1.928242	Normal
GTEX-QLQW-0326-SM-447A8	1.260503	0.712584	1.93247	2.141985	Normal
GTEX-T8EM-0826-SM-4DM76	0.722287	0.291152	1.274015	2.465639	Normal
GTEX-ZZPU-0726-SM-5N9C8	1.58753	0.306293	1.157881	2.04277	Normal
GTEX-ZLWG-0326-SM-4WWC7	1.029394	0.350071	1.262793	2.313115	Normal
GTEX-Y3IK-0426-SM-4WWE2	1.198573	0.645763	1.65091	3.010557	Normal
GTEX-YEC3-0626-SM-4YCFE	1.373287	0.845911	1.453248	2.169382	Normal
GTEX-13N11-0226-SM-5KM3C	1.216571	0.202388	1.283233	3.06014	Normal
GTEX-X4LF-0326-SM-4QAS9	0.871908	1.019607	1.509757	2.267608	Normal
GTEX-13FTW-0526-SM-5IFIP	1.313575	0.154228	1.353281	2.176663	Normal
GTEX-11ILO-1526-SM-5A5KZ	0.777006	0.793138	1.318855	2.688047	Normal
GTEX-11TT1-0326-SM-5LUAY	0.975905	0.31619	1.373871	2.244093	Normal
GTEX-13OVI-0526-SM-5IFFQ	1.818287	0.7354	1.325592	2.191551	Normal
GTEX-145MN-1426-SM-5SI9H	1.265128	0.387953	1.260139	2.311552	Normal
GTEX-XPVG-0326-SM-4B653	0.950781	0.411347	1.383855	2.475625	Normal
GTEX-ZAK1-2326-SM-5CVMY	0.634909	0.930952	0.890012	1.972059	Normal
GTEX-ZDYS-2526-SM-4WKGU	1.559329	0.635428	1.653716	1.899462	Normal
GTEX-XXEK-1726-SM-4BRVB	1.166322	0.312493	1.090834	2.281693	Normal
GTEX-Y8E4-1326-SM-5IFIY	0.734477	0.257139	1.466607	2.109632	Normal
GTEX-ZP4G-0426-SM-4YCER	1.066449	0.309184	1.07471	2.422218	Normal
GTEX-WHSB-0726-SM-4M1XQ	0.870128	0.439483	0.990898	2.509792	Normal

GTEX-146FQ-1526-SM-5Q5CX	1.196479	0.560306	1.340285	2.207463	Normal
GTEX-V955-0326-SM-4JBGV	1.343278	0.686257	0.984093	2.316858	Normal
GTEX-13PL7-2226-SM-5L3IC	1.308494	0.446808	1.638448	2.88144	Normal
GTEX-12WSD-1626-SM-5GCNR	1.417935	0.435533	1.417935	1.829546	Normal
GTEX-12WSK-0226-SM-5BC62	1.729584	0.395933	1.454473	2.346697	Normal
GTEX-Y8LW-1026-SM-5IFJY	1.217305	0.335215	1.065996	2.029967	Normal
GTEX-U8XE-2026-SM-3DB8S	1.022808	0.529723	1.146804	3.208611	Normal
GTEX-13U4I-1526-SM-5IFFF	0.661696	0.609788	1.258053	2.313256	Normal
GTEX-PLZ6-0726-SM-3P619	1.192418	0.71934	1.2502	1.756001	Normal
GTEX-13CF3-1026-SM-5LZWY	0.75097	0.554166	1.207466	2.500846	Normal
GTEX-1211K-1126-SM-5EGGB	1.151738	0.148669	1.670618	2.088227	Normal
GTEX-WHWD-0726-SM-4OORX	0.941537	0.618147	1.377018	2.017452	Normal
GTEX-11I78-0626-SM-5A5LZ	1.253777	0.424467	1.471332	2.275078	Normal
GTEX-W5X1-0226-SM-5CHTO	0.026369	0.026369	0.026369	0.026369	Normal
GTEX-ZVT2-2026-SM-5NQ8Q	1.544056	0.925792	1.262567	2.109412	Normal
GTEX-ZYY3-0826-SM-5E44R	0.487647	0.6941	1.248495	1.860065	Normal
GTEX-OHPL-1026-SM-3MJGI	1.363397	0.705071	1.358111	1.93659	Normal
GTEX-146FR-0126-SM-5Q5F3	0.906487	0.460227	1.886251	1.890527	Normal
GTEX-11EQ9-1026-SM-5H134	1.09472	0.126787	1.228394	2.219463	Normal
GTEX-1399S-0326-SM-5IFFS	1.142692	0.588934	1.229678	1.731033	Normal
GTEX-ZYFG-0826-SM-5BC5T	1.100671	0.600138	0.969073	2.487594	Normal
GTEX-PX3G-1026-SM-48TZW	1.163321	0.402447	1.243409	2.594738	Normal
GTEX-W5WG-0826-SM-4RGNE	1.105858	0.356629	1.230531	2.05631	Normal
GTEX-OOBK-1026-SM-48TC2	1.384032	0.323003	1.188255	2.220164	Normal
GTEX-XBED-0226-SM-47JY8	1.220864	0.27007	1.54033	1.875951	Normal
GTEX-S3XE-0526-SM-4AD4G	1.237433	0.502685	1.470177	1.819382	Normal
GTEX-RM2N-0326-SM-48FD8	0.623436	0.544553	1.242589	1.90636	Normal
GTEX-ZYW4-2126-SM-59HJ9	1.705803	0.520158	1.295744	2.550253	Normal
GTEX-X5EB-0526-SM-46MVP	1.793334	1.906862	1.618195	2.295177	Normal
GTEX-YF7O-1326-SM-4W1ZR	0.670147	0.451205	1.423313	1.845461	Normal
GTEX-132AR-1826-SM-5EGHR	0.995004	0.464093	0.911421	2.431507	Normal
GTEX-131XE-1926-SM-5IFER	0.620323	0.83095	1.406704	3.017111	Normal
GTEX-Q2AI-0426-SM-48U13	0.910502	0.416987	1.531729	2.269624	Normal
GTEX-QESD-0226-SM-447BH	0.498096	0.302846	1.757814	3.124707	Normal
GTEX-WH7G-0826-SM-4LVMR	0.781883	0.163843	1.462215	1.984729	Normal
GTEX-WFON-0626-SM-4LVLX	1.023998	0.881495	1.633208	2.862943	Normal
GTEX-ZYWO-1326-SM-5SI8X	0.724381	0.649478	1.231562	1.961832	Normal
GTEX-WFJO-0626-SM-4LVMC	1.141302	0.71326	1.309586	2.287354	Normal
GTEX-QV31-0226-SM-447BO	1.221357	0	1.283996	1.66858	Normal
GTEX-S4P3-0626-SM-4AD59	1.061523	1.080854	1.554567	2.363786	Normal
GTEX-WYVS-0926-SM-4SOJV	1.069123	0.543398	1.226907	2.071929	Normal
GTEX-YEC4-1326-SM-5IFHG	1.14568	0.477496	1.73817	1.720722	Normal
GTEX-ZEX8-1026-SM-4WKHE	0.78798	0.521796	1.225404	2.50949	Normal
GTEX-111YS-1226-SM-5EGGJ	1.061199	0.427988	1.478906	2.092947	Normal

GTEX-SIU7-0926-SM-4BRX1	0.681721	0.672577	1.35009	1.787337	Normal
GTEX-13FH7-1426-SM-5IFIC	1.367475	0.499779	1.217736	1.95084	Normal
GTEX-WFG8-0326-SM-4LVN4	0.784616	0.711064	1.52508	2.383021	Normal
GTEX-XYKS-1226-SM-4BRVI	2.179437	0.656721	1.531893	2.803404	Normal
GTEX-144FL-1526-SM-5Q5CA	0.930949	0.746823	0.911296	2.802766	Normal
GTEX-R53T-0426-SM-48FEM	0.859528	0.597305	1.381986	2.473129	Normal
GTEX-QDVJ-1226-SM-48U1V	0.989967	1.066054	1.500958	2.607611	Normal
GTEX-Y3I4-0826-SM-4TT2A	0.777951	0.629134	1.1886	1.97581	Normal
GTEX-Y9LG-0726-SM-4VDS3	1.396723	0.837827	1.451787	3.257367	Normal
GTEX-Q2AH-0926-SM-48TZK	1.565088	1.400172	1.386516	2.343141	Normal
GTEX-R55G-0326-SM-48FDM	1.215467	0.437534	1.358674	2.798045	Normal
GTEX-11ONC-0526-SM-5BC57	0.851067	0.43042	1.062697	1.909571	Normal
GTEX-1399U-0926-SM-5IFHA	1.032047	0.35997	1.313251	2.041481	Normal
GTEX-WRHK-0226-SM-4MVOH	1.305589	0.368083	1.55506	2.052009	Normal
GTEX-11LCK-0226-SM-5A5M6	1.098262	0.305807	0.971805	1.857518	Normal
GTEX-117YX-0226-SM-5EGH6	0.702752	0.829327	1.217736	2.517483	Normal
GTEX-X15G-0726-SM-4PQZ5	0.682984	0.62158	1.489672	2.297514	Normal
GTEX-144GN-0826-SM-5LU5G	1.278221	0.329002	1.290217	2.404535	Normal
GTEX-OHPM-1026-SM-3LK74	1.018636	0.707131	1.188109	2.035968	Normal
GTEX-PW2O-0826-SM-48TC5	1.002273	0.256448	1.070582	2.222673	Normal
GTEX-QV44-0426-SM-4R1KF	0.914202	0.262667	1.042406	2.088955	Normal
GTEX-P4PP-1026-SM-3NM9O	0.86092	0.539414	1.403656	1.88137	Normal
GTEX-QCQG-0426-SM-48U29	1.40034	0.534915	1.641709	2.156241	Normal
GTEX-ZPIC-0926-SM-4WWFK	1.477228	0.558838	1.15789	2.134876	Normal
GTEX-ZT9W-0926-SM-57WFS	0.949544	0.505844	1.440999	1.95045	Normal
GTEX-11VI4-0426-SM-5EGHZ	1.560328	1.156011	1.39348	2.567046	Normal
GTEX-Y5V5-1026-SM-5LUAH	1.304888	0.419965	1.385169	2.130012	Normal
GTEX-VUSG-0326-SM-3GIJ7	0.757945	0.538103	1.277205	2.5537	Normal
GTEX-S95S-0726-SM-4B64H	0.705914	0.867011	1.249512	2.532331	Normal
GTEX-WYJK-2426-SM-4ONDQ	1.108479	1.130922	1.368093	2.074708	Normal
GTEX-U8XE-2026-SM-5CHQF	0.026388	0.026388	0.026388	0.026388	Normal
GTEX-13YAN-2126-SM-5Q5C4	1.515155	0.371791	1.49263	2.34412	Normal
GTEX-13VXT-1226-SM-5LU3M	1.398541	1.02496	1.233128	2.684332	Normal
GTEX-S32W-0826-SM-4AD5Z	1.43134	0.658168	1.155164	1.928897	Normal
GTEX-ZG7Y-0326-SM-4WWEY	0.933798	1.003407	1.416504	2.039212	Normal
GTEX-YB5K-1826-SM-5IFJC	0.951912	0.369301	1.192807	1.814553	Normal
GTEX-111CU-0526-SM-5EGHK	1.101562	0.33015	1.069877	2.262231	Normal
GTEX-13O61-2126-SM-5IJEO	1.132557	0.376238	0.990759	2.109362	Normal
GTEX-14ICL-1126-SM-5S2RE	1.323252	0.913832	1.239264	2.826315	Normal
GTEX-12WSL-0426-SM-5GCNX	1.149278	0.69424	1.276719	1.910895	Normal
GTEX-14AS3-0326-SM-5Q5DB	0.866889	0.27534	1.427613	2.27004	Normal
GTEX-132QS-0926-SM-5P9GB	1.242331	0.55773	1.842675	2.223457	Normal
GTEX-13D11-2226-SM-5IFEO	0.905963	0.919736	1.263064	1.978249	Normal
GTEX-ZDTT-1126-SM-4WKFW	1.658761	0.292532	1.499497	2.128576	Normal

GTEX-PSDG-1526-SM-48TCY	0.804153	0.564187	1.42262	1.972834	Normal
GTEX-13NZA-1726-SM-5J1NA	2.490473	1.921708	2.134076	3.307428	Normal
GTEX-ZTPG-1026-SM-5DUWP	1.036051	1.058884	1.223139	3.007742	Normal
GTEX-ZF29-1126-SM-4WKGO	1.058605	0.480845	1.569072	2.121916	Normal
GTEX-11P7K-0526-SM-5BC5I	1.320076	0.478009	1.353306	2.286996	Normal
GTEX-ZVZP-0626-SM-59HL5	0.986382	0.57229	1.58114	3.009634	Normal
GTEX-146FH-1826-SM-5QGQ7	2.348115	1.275258	0.994141	2.093864	Normal
GTEX-XMK1-0326-SM-4B652	1.058274	0.384489	1.268932	2.163236	Normal
GTEX-13FTX-1226-SM-5IFGN	1.377714	0.301217	1.247396	2.198806	Normal
GTEX-TMMY-1326-SM-4DXU9	1.059516	0.294991	1.352924	2.071707	Normal
GTEX-12WSN-0826-SM-5GCOF	1.361669	0.78913	1.347424	1.987143	Normal
GTEX-ZYT6-1326-SM-5E453	1.30196	0.247108	0.918368	2.41861	Normal
GTEX-WXYG-0826-SM-4ONC7	1.69946	0.453407	1.703891	2.589281	Normal
GTEX-11NSD-0526-SM-5A5LT	0.794361	0.286584	1.230079	1.927739	Normal
GTEX-WI4N-1826-SM-4OOSF	0.763305	0.833749	1.092396	2.667249	Normal
GTEX-SNOS-0926-SM-4DM7A	0.930949	1.064282	1.247396	2.501595	Normal
GTEX-1122O-0726-SM-5GIEV	0.893969	0.26852	1.519146	2.045678	Normal
GTEX-WHPG-0326-SM-4M1XV	0.696004	0.467445	1.268768	2.40101	Normal
GTEX-X3Y1-0726-SM-3P5YU	1.297929	0.744412	1.125728	1.721832	Normal
GTEX-ZC5H-0826-SM-5N9FH	0.976035	0.915075	1.3448	2.87704	Normal
GTEX-13X6H-0626-SM-5LU53	1.370511	0.347237	1.213848	2.766293	Normal
GTEX-SE5C-0326-SM-4BRWX	0.858471	0.333661	1.090693	2.435947	Normal
GTEX-1128S-0826-SM-5GZZI	0.772878	0.707567	1.158325	2.414958	Normal
GTEX-YEC3-0626-SM-5IFHZ	0.987899	0.777689	1.491025	2.017223	Normal
GTEX-NPJ8-2126-SM-3MJGK	0.849919	0.981981	0.796304	2.365746	Normal
GTEX-R55D-1426-SM-48FEN	0.979379	0.160714	1.368404	2.225127	Normal
GTEX-ZAB5-0826-SM-5P9FU	1.17271	0.307026	1.430067	2.619877	Normal
GTEX-ZF2S-0426-SM-4WKGP	1.157142	0.445259	1.50224	2.300046	Normal
GTEX-XQ8I-1926-SM-4BOOK	0.895738	0.35924	0.740072	1.952491	Normal
GTEX-SUCS-1426-SM-4DM5W	1.157142	0.320505	1.217051	1.890167	Normal
GTEX-1339X-1026-SM-5IFH5	0.726253	0.659248	0.970906	2.201199	Normal
GTEX-PWOO-0626-SM-48TZH	1.139329	0.477007	1.139329	2.578369	Normal
GTEX-13SLX-1326-SM-5S2QS	0.865008	0.21526	0.853178	1.968983	Normal
GTEX-Y5LM-0526-SM-4V6G3	0.955773	0.392833	1.467428	2.2829	Normal
GTEX-UJHI-0626-SM-3DB8T	0.96949	0.558164	0.876622	2.812134	Normal
GTEX-P4QS-1026-SM-3NMCW	0.914316	0.625742	1.198287	2.149847	Normal
GTEX-139YR-1526-SM-5IFJ1	0.996257	1.506485	1.075111	3.190282	Normal
GTEX-1399R-0426-SM-5IJE3	0.930949	0.348865	1.527348	2.306798	Normal
TCGA-H6-A45N-11A	0.893973	2.03779	1.638448	3.901335	Normal
TCGA-HV-A5A3-11A	1.850968	2.031034	2.068558	5.140083	Normal
TCGA-H6-8124-11A	2.094475	1.558537	2.51977	5.132858	Normal
TCGA-YB-A89D-11A	3.009068	4.651328	2.890659	5.160401	Normal
TCGA-3A-A9IO-01A	4.09777	0.806635	0.347075	3.996474	Tumor
TCGA-US-A774-01A	2.625478	3.711026	2.408264	5.121101	Tumor

TCGA-HZ-A49H-01A	1.874793	2.118372	2.227468	4.279885	Tumor
TCGA-FB-A4P5-01A	2.501096	4.433917	2.369565	4.287219	Tumor
TCGA-FB-AAPS-01A	2.903782	3.257885	2.298455	4.751822	Tumor
TCGA-3A-A9IN-01A	2.182428	3.308485	1.252567	3.847334	Tumor
TCGA-HV-A5A5-01A	2.275149	3.185438	2.281897	4.930094	Tumor
TCGA-H6-8124-01A	3.895456	3.378545	3.801672	5.18633	Tumor
TCGA-US-A77G-01A	3.925767	2.002164	2.906797	5.223762	Tumor
TCGA-3A-A9IJ-01A	2.58563	2.296074	0.354527	3.680475	Tumor
TCGA-HZ-A8P0-01A	3.117572	3.652971	2.222245	3.990754	Tumor
TCGA-IB-7644-01A	3.398698	2.270554	3.343565	5.571276	Tumor
TCGA-IB-A7LX-01A	4.666978	5.323442	4.873795	5.50205	Tumor
TCGA-2J-AABK-01A	2.428121	4.61818	2.279126	4.478249	Tumor
TCGA-HZ-A9TJ-06A	2.80184	1.920641	2.990999	5.361963	Tumor
TCGA-S4-A8RP-01A	2.879792	3.004891	2.587274	4.809687	Tumor
TCGA-2L-AAQM-01A	0.741216	3.064236	0.586274	3.504774	Tumor
TCGA-XD-AAUG-01A	2.482785	2.900506	2.250286	4.776528	Tumor
TCGA-M8-A5N4-01A	3.785203	4.975824	3.181472	5.279856	Tumor
TCGA-2L-AAQI-01A	4.057449	3.388515	3.876706	5.42662	Tumor
TCGA-HZ-7919-01A	4.317382	5.520893	2.881881	5.699944	Tumor
TCGA-HZ-8005-01A	3.917193	6.643575	3.517578	5.374141	Tumor
TCGA-IB-AAUM-01A	1.795096	1.981343	2.13959	4.137554	Tumor
TCGA-IB-7888-01A	2.801049	2.534814	2.709308	4.84669	Tumor
TCGA-2J-AAB9-01A	3.262515	4.492077	3.099148	4.950992	Tumor
TCGA-2L-AAQJ-01A	3.221854	3.854433	2.906797	4.930094	Tumor
TCGA-IB-AAUU-01A	3.722104	4.612701	2.8165	5.579121	Tumor
TCGA-Q3-A5QY-01A	2.235906	5.268856	2.162564	3.730353	Tumor
TCGA-FB-AAQ1-01A	3.208432	6.567624	3.572423	5.638172	Tumor
TCGA-HV-A5A3-01A	4.672702	4.396069	2.644845	5.746756	Tumor
TCGA-IB-AAUO-01A	3.193046	3.811123	2.846477	5.753722	Tumor
TCGA-IB-8127-01A	3.626662	5.970683	3.885033	5.63161	Tumor
TCGA-FB-A545-01A	4.84878	3.812897	3.555927	5.034046	Tumor
TCGA-YH-A8SY-01A	3.211077	3.804959	2.827048	4.826344	Tumor
TCGA-IB-A5SQ-01A	2.805522	3.882664	2.656516	4.62887	Tumor
TCGA-HV-A7OP-01A	2.782727	1.104912	2.121848	3.040357	Tumor
TCGA-FB-AAQ6-01A	3.069741	4.034629	2.022406	4.165739	Tumor
TCGA-FB-AAQ2-01A	5.060292	7.259253	4.716535	5.43948	Tumor
TCGA-US-A77J-01A	1.756388	2.654549	1.483351	4.277676	Tumor
TCGA-F2-7273-01A	2.06445	1.428501	2.359741	5.329953	Tumor
TCGA-3A-A9I9-01A	2.143404	6.481089	3.086956	4.655158	Tumor
TCGA-IB-7654-01A	3.052684	2.344411	2.716362	5.647208	Tumor
TCGA-IB-7893-01A	4.393619	2.763318	4.58207	5.264151	Tumor
TCGA-S4-A8RM-01A	2.962924	0.991038	2.474032	5.153202	Tumor
TCGA-IB-AAUW-01A	1.894807	1.805471	2.520992	5.199524	Tumor
TCGA-FB-AAPZ-01A	3.617604	5.161903	3.38804	5.432148	Tumor

TCGA-IB-AAUS-01A	2.53717	4.561455	2.800628	4.805552	Tumor
TCGA-F2-6879-01A	4.106965	5.756091	3.035168	4.843187	Tumor
TCGA-3A-A9IS-01A	3.194743	1.060731	0.406446	4.506959	Tumor
TCGA-L1-A7W4-01A	3.239319	4.277676	3.197938	6.142067	Tumor
TCGA-FB-A7DR-01A	2.533981	3.723229	2.15583	5.255911	Tumor
TCGA-2J-AABO-01A	4.230289	6.041312	3.507257	4.806548	Tumor
TCGA-3A-A9IH-01A	4.369149	5.109499	3.721534	4.937417	Tumor
TCGA-PZ-A5RE-01A	3.510725	5.956518	2.443141	4.82292	Tumor
TCGA-HZ-8003-01A	2.619624	3.95817	2.339526	4.756154	Tumor
TCGA-3A-A9IB-01A	3.695127	6.005546	3.334527	5.262457	Tumor
TCGA-H6-A45N-01A	3.247922	4.734853	2.264778	5.034046	Tumor
TCGA-HZ-8001-01A	3.006129	4.358913	2.111026	4.434778	Tumor
TCGA-IB-A7M4-01A	3.614988	5.083176	2.914233	5.534873	Tumor
TCGA-2J-AABP-01A	1.62917	4.973302	2.253741	4.81691	Tumor
TCGA-HZ-A77Q-01A	3.268981	5.131315	3.148769	5.308884	Tumor
TCGA-HZ-7926-01A	3.277276	4.612701	3.348215	5.773461	Tumor
TCGA-XN-A8T5-01A	3.22365	5.730363	2.815659	4.747396	Tumor
TCGA-IB-A5ST-01A	2.197709	4.791491	2.305315	4.677457	Tumor
TCGA-Z5-AAPL-01A	1.972968	2.198737	1.491898	4.314309	Tumor
TCGA-IB-7652-01A	2.880189	2.317681	3.832468	4.893089	Tumor
TCGA-IB-7886-01A	3.661998	5.679764	3.640651	5.432148	Tumor
TCGA-FB-AAQ0-01A	3.719905	5.043129	2.914596	4.753915	Tumor
TCGA-IB-7897-01A	2.82579	2.680144	2.948356	5.202445	Tumor
TCGA-2J-AABH-01A	3.860734	1.812474	3.659955	4.77012	Tumor
TCGA-IB-AAUT-01A	2.243001	1.67262	2.202679	5.076289	Tumor
TCGA-HV-A5A4-01A	2.617556	1.271216	2.321421	4.9658	Tumor
TCGA-US-A779-01A	3.404851	2.282271	2.643133	4.677457	Tumor
TCGA-FB-AAPP-01A	3.870301	2.003239	2.638441	4.811946	Tumor
TCGA-HV-A5A6-01A	3.016374	3.998938	3.021418	4.751822	Tumor
TCGA-IB-7887-01A	3.011614	2.688047	3.1153	5.125473	Tumor
TCGA-FB-A78T-01A	2.618409	2.948356	2.090453	5.184944	Tumor
TCGA-IB-7646-01A	3.619675	5.34651	2.786801	5.936701	Tumor
TCGA-RL-AAAS-01A	1.851327	2.734055	2.060349	4.55679	Tumor
TCGA-US-A77E-01A	3.180555	4.376987	2.273517	4.935028	Tumor
TCGA-HZ-8636-01A	3.894247	2.77102	3.068042	4.837601	Tumor
TCGA-IB-AAUP-01A	2.862387	4.329415	2.451695	4.727671	Tumor
TCGA-IB-7885-01A	3.362197	4.562319	2.444306	5.662233	Tumor
TCGA-IB-AAUR-01A	2.227896	2.06445	2.233616	4.516367	Tumor
TCGA-IB-7889-01A	3.44766	4.157443	3.724847	4.560504	Tumor
TCGA-F2-A8YN-01A	3.951863	5.223762	3.246001	5.189188	Tumor
TCGA-3E-AAAZ-01A	4.302269	2.112493	2.579901	4.839831	Tumor
TCGA-HZ-8637-01A	1.597986	2.563675	2.41667	4.72967	Tumor
TCGA-HZ-A4BH-01A	3.844328	5.459405	2.43161	5.240536	Tumor
TCGA-3A-A9I5-01A	1.966158	2.833523	1.136047	3.698414	Tumor

TCGA-XD-AAUL-01A	3.638476	5.888125	2.045437	5.249649	Tumor
TCGA-HZ-7923-01A	2.064834	3.022241	2.387833	4.844319	Tumor
TCGA-2J-AABE-01A	3.626181	4.737865	2.750066	4.600562	Tumor
TCGA-FB-AAPU-01A	2.597408	3.268049	1.925044	5.284877	Tumor
TCGA-3A-A9IC-01A	3.212803	3.499592	2.076907	4.851972	Tumor
TCGA-2J-AABI-01A	2.468133	1.330767	3.740343	6.602984	Tumor
TCGA-HZ-A77O-01A	3.445286	4.156078	3.017195	4.327137	Tumor
TCGA-YY-A8LH-01A	3.25842	4.506959	2.151657	4.27251	Tumor
TCGA-3E-AAAY-01A	1.977097	2.924602	2.35513	4.646488	Tumor
TCGA-3A-A9IZ-01A	3.643759	3.537974	2.236283	5.610095	Tumor
TCGA-IB-A6UG-01A	3.312597	3.836552	3.32369	4.270333	Tumor
TCGA-FB-A5VM-01A	3.511229	4.765682	3.027868	5.962054	Tumor
TCGA-RB-AA9M-01A	3.047129	5.098493	2.696022	5.183563	Tumor
TCGA-HZ-A49G-01A	2.677835	3.337944	3.178734	5.10123	Tumor
TCGA-HV-AA8X-01A	3.971312	3.396261	2.265562	4.691956	Tumor
TCGA-LB-A7SX-01A	3.993331	4.982332	3.150931	5.28823	Tumor
TCGA-HZ-7922-01A	3.929381	5.842695	2.919958	5.706822	Tumor
TCGA-2J-AABV-01A	1.633913	2.230369	1.540116	3.497993	Tumor
TCGA-IB-AAUN-01A	4.415168	4.873795	4.120036	4.986089	Tumor
TCGA-2L-AAQA-01A	4.642468	3.900205	2.634065	4.77012	Tumor
TCGA-IB-A6UF-01A	4.172525	6.191498	2.537571	5.763574	Tumor
TCGA-HV-A7OL-01A	3.123715	6.041312	2.153222	4.428245	Tumor
TCGA-IB-7651-01A	2.298455	4.067835	2.228732	4.773396	Tumor
TCGA-2J-AABU-01A	4.914708	5.470363	3.809986	5.761043	Tumor
TCGA-XN-A8T3-01A	3.405344	4.647474	3.237595	4.702781	Tumor
TCGA-HV-AA8V-01A	3.408634	4.43739	2.674278	5.329953	Tumor
TCGA-S4-A8RO-01A	4.279885	4.864553	3.176128	5.265615	Tumor
TCGA-IB-8126-01A	1.763117	2.16335	2.325628	4.833038	Tumor
TCGA-2J-AABF-01A	2.311956	4.101057	2.35324	5.099847	Tumor
TCGA-US-A776-01A	3.348616	2.097505	1.439417	3.894854	Tumor
TCGA-2J-AABT-01A	2.296074	3.91126	2.304564	5.15173	Tumor
TCGA-IB-7891-01A	2.593121	1.733123	2.811628	5.264151	Tumor
TCGA-XD-AAUI-01A	3.464554	5.959294	2.966342	5.135774	Tumor
TCGA-RB-A7B8-01A	2.546902	2.909235	2.226696	4.128179	Tumor
TCGA-XD-AAUH-01A	2.58885	2.134008	2.058489	4.654235	Tumor
TCGA-2J-AAB6-01A	4.330227	6.116163	3.514185	5.837696	Tumor
TCGA-2J-AAB4-01A	2.809245	4.75073	2.460717	5.214673	Tumor
TCGA-FB-A4P6-01A	2.30493	3.480759	2.398951	4.926604	Tumor
TCGA-IB-AAUV-01A	2.019775	2.233262	2.460717	4.941188	Tumor
TCGA-3A-A9IL-01A	1.976743	2.281897	0.887036	3.473398	Tumor
TCGA-IB-AAUQ-01A	3.666422	7.191424	2.345171	4.468693	Tumor
TCGA-FB-AAPQ-01A	3.391953	4.533235	2.267806	4.810878	Tumor
TCGA-IB-A5SS-01A	4.165739	6.412564	3.585009	5.877204	Tumor
TCGA-IB-7645-01A	2.377294	2.937133	3.19656	5.408899	Tumor

TCGA-2J-AAB1-01A	3.146554	3.683785	2.830206	4.935028	Tumor
TCGA-IB-A5SP-01A	4.761917	4.68046	3.391413	5.401318	Tumor
TCGA-OE-A75W-01A	2.639638	5.500247	1.996774	4.043513	Tumor
TCGA-F2-7276-01A	2.381557	3.059065	2.291815	5.534873	Tumor
TCGA-3A-A9IX-01A	2.846099	3.362679	2.903362	4.960759	Tumor
TCGA-3A-A9IV-01A	2.650664	1.302802	1.614385	4.545724	Tumor
TCGA-HZ-8315-01A	3.5823	5.313517	3.464019	5.488978	Tumor
TCGA-Q3-AA2A-01A	3.425663	4.241259	2.623129	4.239725	Tumor
TCGA-HZ-7925-01A	2.577104	4.992365	2.711314	5.189188	Tumor
TCGA-HZ-7289-01A	3.998938	6.050856	2.340703	4.726725	Tumor
TCGA-HZ-8519-01A	3.024841	2.004028	1.743492	4.55679	Tumor
TCGA-F2-A44G-01A	3.343565	4.791491	2.548029	5.041841	Tumor
TCGA-HZ-8638-01A	2.303424	3.315899	2.025748	5.532929	Tumor
TCGA-3A-A9IU-01A	3.837789	4.931323	3.041226	5.976718	Tumor
TCGA-2J-AABR-01A	2.264778	2.755263	2.230585	4.986089	Tumor
TCGA-2J-AABA-01A	3.023526	4.298544	2.800628	5.032674	Tumor
TCGA-F2-6880-01A	1.155155	1.049528	1.934437	3.936631	Tumor
TCGA-F2-A7TX-01A	2.490979	3.745934	2.301916	5.196598	Tumor
TCGA-HZ-A4BK-01A	3.035168	5.22972	2.601016	5.095862	Tumor
TCGA-HZ-A77P-01A	3.010345	3.763107	2.460717	4.970881	Tumor
TCGA-3A-A9J0-01A	3.83072	2.023951	2.64859	4.893089	Tumor
TCGA-HZ-A8P1-01A	2.983525	3.914864	1.931464	4.550223	Tumor
TCGA-2L-AAQL-01A	3.200125	2.668756	1.789255	4.775517	Tumor
TCGA-HZ-8317-01A	2.524193	4.230289	2.589191	4.996433	Tumor
TCGA-HZ-7920-01A	2.656516	3.008632	1.576529	4.840842	Tumor
TCGA-HZ-A49I-01A	2.900101	3.990083	3.818306	4.873795	Tumor
TCGA-HZ-A9TJ-01A	2.696756	1.968342	2.871049	4.926604	Tumor
TCGA-YB-A89D-01A	3.667036	4.375428	2.932974	5.187773	Tumor
TCGA-IB-7649-01A	2.636839	3.168553	3.01247	4.958249	Tumor
TCGA-LB-A8F3-01A	1.253852	1.586961	1.902671	4.185835	Tumor
TCGA-2J-AAB8-01A	2.930041	4.825224	2.878139	4.786177	Tumor
TCGA-3A-A9IR-01A	3.309859	1.406674	1.066449	4.234619	Tumor
TCGA-2L-AAQE-01A	4.536719	4.351104	2.505778	5.746756	Tumor
TCGA-3A-A9I7-01A	3.019359	4.75814	2.767	5.122629	Tumor
TCGA-IB-7890-01A	4.222471	5.895988	5.044472	5.208728	Tumor
TCGA-LB-A9Q5-01A	3.110539	4.440648	2.366204	4.714309	Tumor
TCGA-HZ-7924-01A	2.656107	1.457704	2.366929	5.408899	Tumor
TCGA-HZ-8002-01A	2.607308	3.182819	3.101709	5.361963	Tumor
TCGA-FB-AAPY-01A	2.370395	4.581104	2.708892	4.515525	Tumor
TCGA-F2-A44H-01A	2.048467	2.916685	3.04078	4.641438	Tumor
TCGA-IB-A5SO-01A	2.746847	3.343119	2.067164	5.205482	Tumor
TCGA-FB-AAQ3-01A	3.380974	4.75291	3.403919	4.259284	Tumor
TCGA-HZ-7918-01A	1.710906	1.553835	3.056835	5.390506	Tumor
TCGA-H8-A6C1-01A	2.64859	3.256497	2.336134	4.827411	Tumor
