# Supplementary material for: Circadian Genes MBOAT2/CDA/LPCAT2/B4GALT5 in the Metabolic Pathway Serve as New Biomarkers of PACA Prognosis and Immune Infiltration
Source: Life (Basel). 2023 Apr 30;13(5):1116. doi: 10.3390/life13051116 (PMC10221058; doi:10.3390/life13051116)
Supplement: Supplementary file 1 [file life-13-01116-s001.zip › Table S3.pdf]

Table S3. The clinical data of PACA patients

Id	futime	fustat	age	gender	grade	stage	T	N
TCGA-XN-A8T5	720	0	53	0	2	1	2	0
TCGA-IB-A5SQ	219	1	56	0	2	1	2	0
TCGA-RL-AAAS	9	0	60	0	2	1	2	0
TCGA-IB-AAUN	144	1	74	0	2	1	2	0
TCGA-XN-A8T3	951	0	67	1	2	1	2	0
TCGA-IB-7890	598	1	73	1	3	1	2	0
TCGA-FB-A5VM	449	0	74	1	3	1	2	0
TCGA-Z5-AAPL	467	0	74	0	1	2	3	0
TCGA-OE-A75W	267	1	75	1	1	2	3	0
TCGA-M8-A5N4	584	0	48	0	2	2	3	0
TCGA-IB-7888	1332	1	66	0	2	2	3	0
TCGA-F2-A44H	586	0	65	1	2	2	3	0
TCGA-2J-AABE	676	0	73	1	2	2	3	0
TCGA-2J-AAB6	293	1	75	1	2	2	3	0
TCGA-F2-A8YN	517	0	76	1	2	2	3	0
TCGA-IB-A5SP	482	0	77	1	2	2	3	0
TCGA-FZ-5922	1101	1	81	1	2	2	3	0
TCGA-2J-AABI	969	0	55	0	3	2	3	0
TCGA-2J-AABR	438	0	60	0	3	2	3	0
TCGA-2J-AABH	677	0	61	1	3	2	3	0
TCGA-IB-7893	117	1	64	1	3	2	3	0
TCGA-HZ-A9TJ	603	0	70	1	2	4	3	0
TCGA-HZ-8636	545	1	58	0	3	4	3	0
TCGA-3A-A9IH	1021	0	66	0	2	1	1	0
TCGA-3A-A9IX	1037	0	40	1	2	1	1	0
TCGA-FB-AAQ6	244	1	85	1	2	1	1	0
TCGA-HZ-8519	454	0	73	1	3	1	1	0
TCGA-3A-A9IV	1103	0	59	0	1	1	1	0
TCGA-3A-A9IR	1542	0	64	0	1	1	1	0
TCGA-3A-A9IJ	1854	0	65	1	1	1	2	0
TCGA-3A-A9IS	998	0	67	1	1	1	2	0
TCGA-HZ-A8P1	7	0	81	1	1	1	2	0
TCGA-HZ-7920	236	1	71	1	2	1	2	0
TCGA-LB-A8F3	379	0	64	0	1	2	2	0
TCGA-FB-AAPU	381	1	41	0	2	2	3	0
TCGA-HZ-8315	299	1	54	0	2	2	3	0
TCGA-HZ-7924	840	0	60	0	2	2	3	0
TCGA-HV-A5A4	232	0	72	0	2	2	3	0
TCGA-H8-A6C1	671	0	53	1	2	2	3	0
TCGA-XD-AAUL	405	0	56	1	2	2	3	0
TCGA-US-A776	844	0	61	1	2	2	3	0
TCGA-HZ-7923	314	0	65	1	2	2	3	0

TCGA-3A-A9I9	634	1	67	1	2	2	3	0
TCGA-HV-A7OL	252	0	70	1	2	2	3	0
TCGA-3E-AAAZ	2182	1	71	1	2	2	3	0
TCGA-FB-AAQ0	473	1	68	1	3	2	3	0
TCGA-2L-AAQJ	394	1	49	0	2	3	4	0
TCGA-HZ-8001	706	0	69	1	2	3	4	0
TCGA-IB-AAUT	287	0	65	1	1	2	2	1
TCGA-2J-AAB4	729	0	48	1	2	2	2	1
TCGA-IB-7654	476	1	80	1	2	2	2	1
TCGA-IB-AAUM	8	0	76	1	3	2	2	1
TCGA-FZ-5924	480	1	83	1	3	2	3	1
TCGA-IB-7645	1502	1	44	0	1	2	3	1
TCGA-IB-7891	648	0	49	0	1	2	3	1
TCGA-2J-AAB9	627	1	70	0	1	2	3	1
TCGA-IB-7889	481	1	85	0	1	2	3	1
TCGA-F2-7276	216	1	64	1	1	2	3	1
TCGA-IB-AAUR	338	0	67	1	1	2	3	1
TCGA-F2-6880	295	0	70	1	1	2	3	1
TCGA-IB-7652	724	0	49	0	2	2	3	1
TCGA-HZ-7919	593	1	52	0	2	2	3	1
TCGA-IB-7897	486	1	53	0	2	2	3	1
TCGA-XD-AAUH	395	0	57	0	2	2	3	1
TCGA-HV-A5A5	289	0	61	0	2	2	3	1
TCGA-IB-7887	110	1	62	0	2	2	3	1
TCGA-IB-A5ST	635	0	64	0	2	2	3	1
TCGA-IB-7651	603	1	64	0	2	2	3	1
TCGA-FB-A78T	375	1	71	0	2	2	3	1
TCGA-2J-AABT	319	0	72	0	2	2	3	1
TCGA-FB-A545	385	0	72	0	2	2	3	1
TCGA-IB-7649	467	1	73	0	2	2	3	1
TCGA-IB-7885	977	0	78	0	2	2	3	1
TCGA-F2-A44G	233	1	79	0	2	2	3	1
TCGA-IB-AAUS	225	0	84	0	2	2	3	11
TCGA-IB-7647	666	1	41	1	2	22	3	1
TCGA-2J-AABO	440	0	43	1	2	2	3	1
TCGA-IB-AAUV	404	0	49	1	2	2	3	1
TCGA-IB-AAUQ	183	1	50	1	2	2	3	1
TCGA-2J-AABA	607	1	55	1	2	2	3	1
TCGA-2L-AAQE	684	1	56	1	2	2	3	1
TCGA-F2-6879	334	1	57	1	2	2	3	1
TCGA-IB-8127	522	0	59	1	2	2	3	1
TCGA-IB-7646	145	1	60	1	2	2	3	1
TCGA-IB-A6UF	666	0	63	1	2	2	3	1
TCGA-IB-AAUP	431	0	68	1	2	2	3	1

TCGA-2J-AABK	484	0	71	1	2	2	3	1
TCGA-IB-A5SO	365	1	71	1	2	2	3	1
TCGA-L1-A7W4	278	1	48	0	3	2	3	1
TCGA-H6-8124	392	0	56	0	3	2	3	1
TCGA-IB-AAUW	230	1	63	0	3	2	3	1
TCGA-IB-AAUO	239	1	64	0	3	2	3	1
TCGA-IB-A5SS	460	1	64	0	3	2	3	1
TCGA-IB-AAUU	245	0	35	1	3	2	3	1
TCGA-FZ-5920	61	1	52	1	3	2	3	1
TCGA-F2-7273	360	0	54	1	3	2	3	1
TCGA-2J-AABU	277	1	56	1	3	2	3	1
TCGA-IB-A6UG	41	1	65	1	33	2	3	1
TCGA-2J-AAB1	66	1	65	1	3	2	3	1
TCGA-2J-AAB8	80	0	71	1	3	2	3	1
TCGA-HZ-7918	969	0	72	1	3	2	3	1
TCGA-2J-AABF	691	1	73	1	3	2	3	1
TCGA-F2-A7TX	95	1	77	1	3	2	3	1
TCGA-IB-7886	123	1	80	1	3	2	3	1
TCGA-2J-AABP	355	0	58	0	4	2	3	1
TCGA-2J-AABV	652	1	74	1	4	2	3	1
TCGA-FZ-5926	541	1	73	0	2	3	3	1
TCGA-IB-8126	75	0	79	0	1	3	4	1
TCGA-IB-7644	394	1	65	0	2	4	3	1
TCGA-XD-AAUG	420	0	66	0	2	4	3	1
TCGA-FZ-5923	619	1	71	1	1	4	4	1
TCGA-RB-AA9M	286	0	43	1	3	2	1	1
TCGA-HZ-A49G	660	0	58	0	2	2	2	1
TCGA-FB-AAPS	228	0	62	0	2	2	2	1
TCGA-HV-AA8X	532	1	75	0	2	2	2	1
TCGA-HZ-A77O	160	1	77	0	2	2	2	1
TCGA-FB-AAPY	1059	1	71	1	2	2	2	1
TCGA-FB-AAPZ	716	0	54	1	3	2	2	1
TCGA-Q3-AA2A	95	0	64	0	1	1	2	1
TCGA-HZ-7922	4	0	61	0	1	2	3	1
TCGA-HZ-8317	378	1	69	0	1	2	3	1
TCGA-2L-AAQM	1383	0	52	1	1	2	3	1
TCGA-FB-A4P6	363	0	54	1	1	2	3	1
TCGA-HZ-7926	518	1	57	1	1	2	3	1
TCGA-HZ-A77P	330	0	77	1	1	2	3	1
TCGA-HZ-7289	661	1	77	1	1	2	3	1
TCGA-XD-AAUI	366	1	50	0	2	2	3	1
TCGA-HZ-A77Q	33	0	55	0	2	2	3	1
TCGA-3A-A9IC	738	1	61	0	2	2	3	1
TCGA-FB-AAQ3	31	1	65	0	2	2	3	1

TCGA-HZ-8003	596	1	65	0	2	2	3	1
TCGA-HZ-A49H	491	0	68	0	2	2	3	1
TCGA-FB-A4P5	179	1	69	0	2	2	3	1
TCGA-LB-A7SX	393	1	74	0	2	2	3	1
TCGA-S4-A8RO	525	0	75	0	2	2	3	1
TCGA-RB-A7B8	466	1	81	0	2	2	3	1
TCGA-3A-A9I7	1323	0	45	1	2	2	3	1
TCGA-3A-A9IZ	308	1	47	1	2	2	3	1
TCGA-FB-AAQ1	123	1	49	1	2	2	3	1
TCGA-IB-A7LX	250	1	57	1	2	2	3	1
TCGA-Q3-A5QY	416	0	58	1	2	2	3	1
TCGA-YB-A89D	350	0	59	1	2	2	3	1
TCGA-HZ-8002	366	1	61	1	2	2	3	1
TCGA-US-A77G	12	1	64	1	2	2	3	1
TCGA-FB-AAPQ	1130	1	65	1	2	2	3	1
TCGA-HZ-7925	614	1	66	1	2	2	3	1
TCGA-3A-A9J0	743	0	75	1	2	2	3	1
TCGA-2L-AAQA	143	1	76	1	2	2	3	1
TCGA-HZ-A49I	308	1	77	1	2	2	3	1
TCGA-PZ-A5RE	470	1	44	0	2	2	3	1
TCGA-FZ-5919	741	1	59	0	3	2	3	1
TCGA-YY-A8LH	2016	0	61	0	3	2	3	1
TCGA-LB-A9Q5	313	1	63	0	3	2	3	1
TCGA-3A-A9IB	224	1	69	0	3	2	3	1
TCGA-HZ-8637	517	1	76	0	3	2	3	1
TCGA-US-A774	695	1	76	0	3	2	3	1
TCGA-S4-A8RP	702	1	77	0	3	2	3	1
TCGA-FB-AAQ2	153	1	81	0	3	2	3	1
TCGA-H6-A45N	397	1	88	0	3	2	3	1
TCGA-HV-AA8V	920	0	50	1	3	2	3	1
TCGA-3A-A9IU	458	1	65	1	3	2	3	1
TCGA-2L-AAQI	103	1	66	1	3	2	3	1
TCGA-S4-A8RM	646	0	67	1	3	2	3	1
TCGA-3E-AAAY	2285	0	67	1	3	2	3	1
TCGA-FB-AAPP	485	1	71	1	3	2	3	1
TCGA-HZ-A4BK	657	0	72	1	3	2	3	1
TCGA-US-A77E	430	1	73	1	3	2	3	1
